# Diverging selection on body size in specialist terrestrial mammals

**DOI:** 10.1038/s41559-025-02959-2

**Published:** 2026-01-28

**Authors:** Shan Huang, Andrew Morozov, Alison Eyres, Xiang-Yi Li Richter

**Affiliations:** 1 School of Geography, Earth and Environmental Sciences, University of Birmingham, Birmingham, UK; 2 School of Computing and Mathematical Sciences, University of Leicester, Leicester, UK; 3 Department of Zoology, University of Cambridge, Cambridge, UK; 4 Institute of Ecology and Evolution, University of Bern, Bern, Switzerland; 5 Department of Biology, University of Konstanz, Konstanz, Germany

**Keywords:** Evolutionary ecology, Macroecology, Ecological modelling, Biodiversity

## Abstract

Body size is a fundamental organismal trait, affecting a wide variety of physiological and ecological functions. Such relationships are often interactive and nonlinear, forming complex feedbacks. In terrestrial mammals, larger bodies are associated with higher mobility in trade-off with larger absolute resource demand. Here we propose a hypothesis, with support from empirical patterns and a mathematical model, that this trade-off interacts with diet specialization to drive diverging selection on body size because specialists are more efficient resource users and have lower mortality risks at extreme sizes. Our analysis of a global terrestrial mammal species dataset found significantly lower proportions of specialists at intermediate sizes, but higher proportions towards extreme sizes; this pattern also applies to species assemblages in zoographic realms. Our mathematical model of coexistence between equal-sized specialists and generalists shows that specialists of extreme sizes have higher equilibrium frequencies and likelihood of coexistence with generalists at quasi-stability. The combined results support dietary specialization as a key factor for shaping body size diversity. Our work highlights the value of connecting ecology and evolution in understanding the diversity of key traits like body size, and calls for further investigations on the related history of resource distribution and lineage diversification.

## Abstract (web summary)

A comparative analysis of trait data combined with a mathematical model suggests that dietary specialization drives selection towards the smallest and largest body sizes in terrestrial mammals, as generalists outcompete specialists at intermediate sizes.

## Main

Animal body size varies across orders of magnitude, and its variation in space and time represents an essential aspect of biodiversity^1–5^. A deep understanding of how body size diversity is shaped can illuminate fundamental mechanisms of evolution and biodiversity^6–9^. However, with its strong links to many physiological and ecological traits, body size probably evolves in response to a concert of physiological constraints, resource demands and environmental pressures^4–6,10–13^, whose effects are dynamic, interacting and difficult to tease apart. In this study, we combine empirical investigation with mathematical modelling to demonstrate the key role of dietary specialization in the evolution, and particularly the diversification, of animal body size.

Animal species with larger bodies generally require more resources and are able to move over longer distances to forage and therefore maintain larger home ranges^14–18^. Meanwhile, the foraging range is expected to correlate negatively with the degree of specialization, reflected in the variety of resources a taxon can use, based on the classic cost–benefit theory: generalists tend to be outcompeted by specialists for local resources and, thus, need to forage in larger spatial areas^19–22^. In a comparative analysis of terrestrial mammals, this expectation was met only in small-sized species, while the opposite pattern was found in species with large bodies: specialists with large bodies tend to have larger home ranges than generalists in order to support their food demand and dietary requirements^23^. To explain these patterns, we propose a hypothesis in which specialists experience diverging selection on body size, favouring either small bodies that minimize foraging-associated mortality or much larger bodies that support long-distance foraging; by contrast, specialization becomes disadvantageous at intermediate body sizes. Our theory underlying this hypothesis focuses on the variation in coexistence outcome when specialists and generalists are present in the same environment, having overlapping resource preferences. Therefore, we expect the patterns to be stronger within regional assemblages than in evolutional lineages whose morphological and functional diversity tend to be constrained by evolutionary conservatism^24–26^.

We use a classic model system in macroecology—terrestrial mammals—to demonstrate the hypothesized contrast of high proportions of dietary specialists at both ends of the body size spectrum and low proportions near the middle. This pattern is observed empirically across the entire clade globally, as well as within regional assemblages, although additional complexity emerges when the data are analysed by taxonomic order and primary dietary group. Furthermore, we develop mathematical models to illustrate the ecological theory that the smallest- and largest-bodied specialists can coexist at higher frequencies with generalists of their respective sizes, owing to differences between specialists and generalists in resource acquisition rates, the scaling of home-range size with body mass, and body-mass-associated mortality risk. Our findings shed light on the fundamental ecological mechanisms that drive evolution and ultimately produce the diversity of body size seen in our empirical synthesis of terrestrial mammals, and probably in other animals.

## Results

### Empirical evidence of low proportions of specialists among intermediate-sized mammals

Globally, terrestrial mammals in our dataset span seven orders of magnitude in body mass (ranging from 1.75 to 3.9 × 10^6^ g; Supplementary Fig. 1), with values logarithmically transformed for subsequent analyses. For a binary comparison, we consider species consuming one type of food as specialists and all others as generalists (dataset 1; see diet types in Supplementary Fig. 2 and variation across orders in Supplementary Fig. 3). Using a Bayesian multilevel regression model to account for the phylogenetic effect typically shown in body size^27^, we found generalists on average to be slightly larger than specialists (mean posterior difference: coef. = 0.11, with a 5–95% credible interval of [0.021 to 0.019]). However, no unidirectional relationship was observed between the number of diet types and body size, as the credible interval of the coefficient included zero (coef. = −0.29 [−0.63 to 0.003]).

**Fig. 1:**
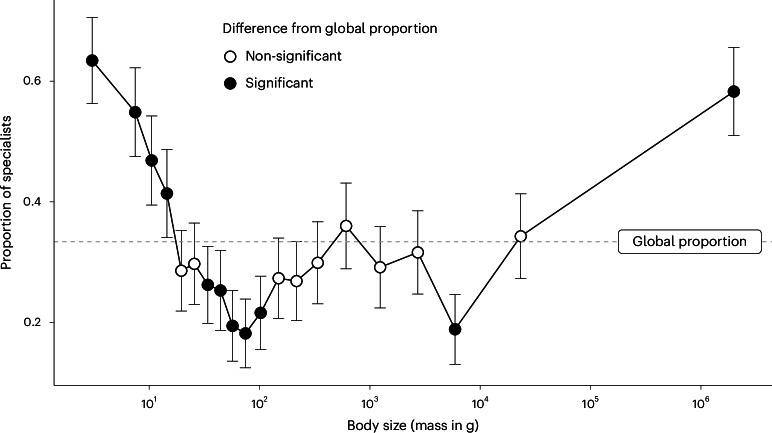
Diverging specialists on the global body size spectrum. The proportion of dietary specialists (points) is high in species with small and large body sizes, but low in species of intermediate size. Species were compared across 20 bins, each representing 5% quantiles of average adult body mass (see data divisions in Supplementary Fig. 1). The error bars represent the 95% binomial proportion confidence intervals. We considered proportions whose confidence intervals do not contain the proportion of specialists in all 3,487 mammal species (33.4%, indicated by the dashed grey line) as significant deviations from the null expectation (solid points). See the text for support from phylogenetic regression models.

When compared across the body size spectrum, the proportions of specialists are higher than the global proportion (33.4%, as the null expectation) in the smallest (<20%) and largest (>95%) quantiles and lower at intermediate sizes (especially quantiles between 30% and 55% and between 85% and 90% (Fig. 1; see consistent patterns with different cut-offs in Supplementary Fig. 4 and a lower-resolution in Supplementary Fig. 5). We also considered the full range of dietary types a species can consume (1–6 out of ten types; Supplementary Fig. 6) using phylogenetic regression models and found that species with a body size further away from the median tended to show dietary specialization, consuming fewer diet types; this was consistently found when we analysed the species smaller (coef. = 0.049 [0.0087 to 0.09]) or larger (coef. = −0.042 [−0.082 to −0.0019]) than the median separately or combined using their absolute difference from the median (coef. = −0.044 [−0.074 to −0.014]).

**Fig. 2:**
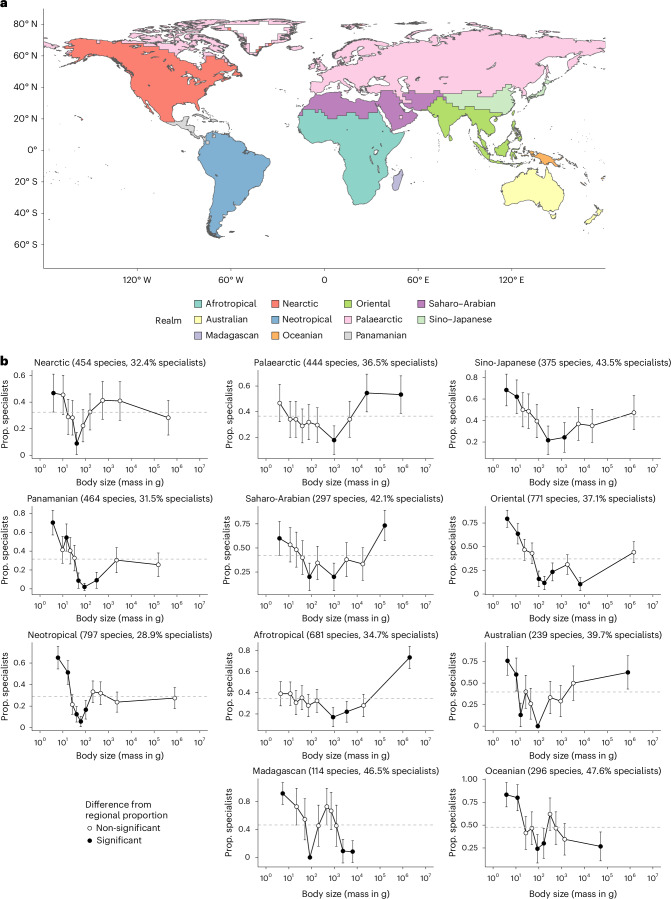
Specialists tend to be of extreme sizes in regional faunas. **a**, Map of all regions, defined according to zoogeographic realms identified by Holt et al.^28^. **b**, The proportion (Prop.) of dietary specialists (points) declines in species of intermediate size. Species were grouped into ten bins, each representing 10% quantiles of average adult body mass (Supplementary Fig. 7). Panel **a** is plotted on the basis of data published in ref. ^28^ (https://macroecology.ku.dk/resources/wallace, accessed on 20 June 2023). In **b**, the total number of species occurring in each region is indicated in the panel titles, and the proportion of specialists in each region (our realm-specific null expectation) is indicated by the dashed grey line. We considered proportions whose 95% binomial proportion confidence intervals (error bars) do not contain the region-specific null as significant deviations (solid points). In most regions, the proportion of specialists in both small and large size bins is relatively high; however, in Oceanian and Madagascan regions—primarily composed of island faunas—the largest size bins show significantly lower proportions of specialists than expected. See results from phylogenetic regression models in Supplementary Table 1.

The low proportion of specialists is consistently found for at least one of the intermediate-sized quantile bins in all regional assemblages (defined on the basis of the zoographic realms from Holt et al.^28^; Fig. 2), although the absolute position of the lowest point varies across regions. In some regions, the proportions of specialists at either the smallest (for example, the Neotropical and Oriental realms) or the largest sizes (for example, the Afrotropical realm) are similar to overall proportions in the respective realms (defined by our region-specific null expectation; see similarly complex patterns from phylogenetic regression models in Supplementary Table 1). However, only in the two island-dominated realms, the Oceanian and Madagascan, does the proportion of specialists drop below the region-specific null for the largest body sizes. Variation in regional patterns was also found in the body size range and frequency distribution (Supplementary Table 2 and Supplementary Fig. 7).

**Fig. 3:**
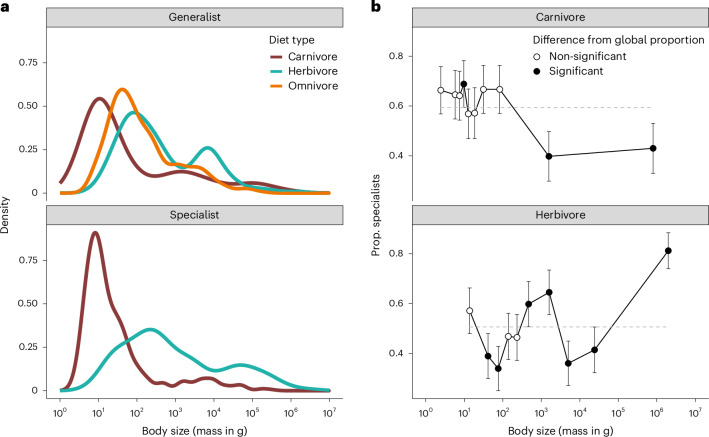
Body size distribution in carnivore and herbivore species. **a**,**b**, Mammals of carnivorous and herbivorous diets (as primary diet types) show different density distributions across the body size spectrum (**a**) and the proportions of specialists deviated from the global patterns (**b**). In **b**, proportions are considered as significantly deviating from the null expectation (solid points) if their 95% binomial proportion confidence intervals (error bars) do not contain the proportion of specialists in all mammal species of the same primary diet type (out of 931 carnivore species and 1,113 herbivore species; indicated by the dashed grey lines). See Supplementary Fig. 11 for additional analyses in which omnivores were included as generalists for both carnivores and herbivores.

Using the global dataset, we further performed two sets of analyses and found complex patterns of body size in relation to specialization within (a) major subclades (taxonomic orders) and (b) the primary dietary categories. Both factors are intrinsically linked to animal ecology and evolution (for example, in evolutionary^27,29^ and metabolic theories^30,31^), but neither is confined to coexisting species even at macroecological scales. The five most species-rich orders (>200 species), Rodentia, Chiroptera, Carnivora, Primates and Soricomorpha, all have world-wide distributions across most zoographic realms (Supplementary Fig. 8). There is a tendency for species of non-extreme size to be generalists, while some of the largest species tend to be specialists (Supplementary Fig. 9; see size distributions in Supplementary Fig. 10), but the only trend detected in regression analyses was for Rodentia (coef. = −0.51 [−0.096 to −0.008]). We also categorized all species in our global dataset into more broadly defined, primary dietary types: carnivores (eating any type of animals, including scavengers; Supplementary Fig. 2; *N* = 963), herbivores (eating plant materials including fruits, nectar and seeds; *N* = 1,172) and omnivores (eating both animal and plant materials; *N* = 1,352). By our binary definition of specialist and generalist above, the omnivores are all generalists while the carnivores and herbivores can be specialists or generalists, so we mainly compared the body size patterns within carnivores and herbivores (see comparison involving omnivores in Supplementary Fig. 11). The largest herbivores are more likely to be specialists, whereas in carnivores, the proportion of specialists is relatively stable in small-to-medium sizes but declines towards the largest sizes (Fig. 3b). Our phylogenetic regression models detected an increase in body size with the number of diet types only in carnivore species smaller than the carnivore median size (coef. = 0.15 [0.0056 to 0.29]). We discuss the complexity of these links to mammalian behaviours and propose directions for further investigation below.

### Mathematical model shows small- or large-sized specialists coexist better with generalists

**Fig. 4:**
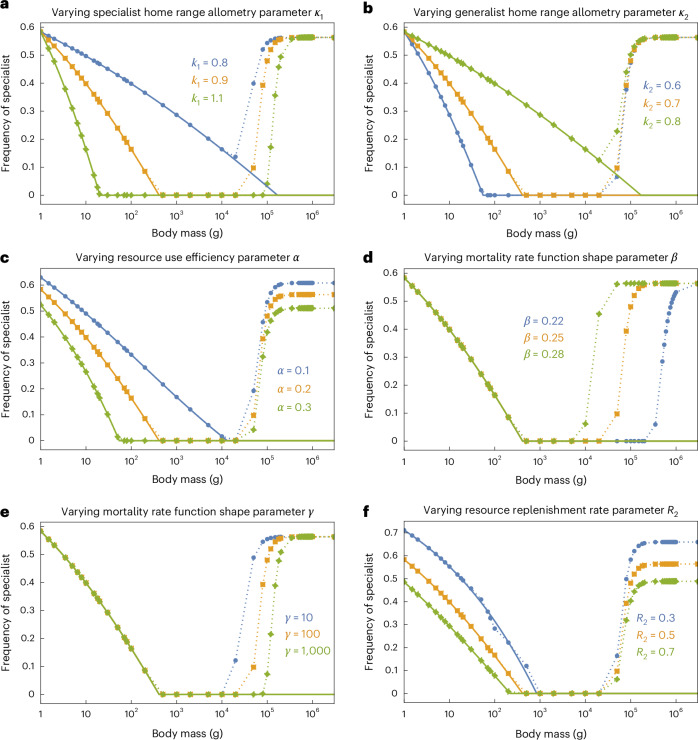
Specialists tend to have higher frequencies at extreme sizes at quasi-stability. Frequency of the specialist consumer at *t* = 10^3^, by which point the competition dynamics for body size ranges up to approximately 10^4^ g have reached equilibrium, while the competition between larger-bodied consumers proceeds slowly in quasi-stability (compared with the frequencies at times *t* = 10^4^, 10^5^ and 10^6^ in Supplementary Figs. 14–16, respectively; see Fig. 5 for examples of competition trajectories). **a**–**f**, Varying model parameters (as specified in the panel headings) produced consistent patterns. The colours represent the different parameters examined in each panel. Symbols connected by dotted lines are numerically generated, which, at equilibrium, are overlaid by the solid lines of the same colours representing analytical solutions of the competition equilibrium. Unless specified otherwise, the parameter values are *k*_1_ = 0.9, *k*_2_ = 0.7, *α* = 0.2, *β* = 0.25, *γ* = 100, *g* = 1, *κ* = 100, *R*_1_ = 1 and *R*_2_ = 0.5.

The results of our model are consistent with the empirical findings (Fig. 1): at the extremes of the body size range of terrestrial mammals, specialists are more likely to coexist with generalists of the same body size (mass) and have higher frequencies (defined by the relative abundance; Fig. 4) than at intermediate sizes. The pattern is robust across broad ranges of parameters during transient competition dynamics (Supplementary Figs. 12 and 13) and after the systems have reached quasi-stability (Supplementary Figs. 14–16).

Our model considers a system of two resources *r*_1_ and *r*_2_ and two consumer species *x*_1_ and *x*_2_, where consumer *x*_1_ is a specialist, only consuming resource *r*_1_ at rate *g*, and *x*_2_ is a generalist consuming both resources at a rate *αg*, with 0 < *α* < 1, thus less efficiently than the specialist. The temporal dynamics of the abundances for the resources and consumers can be described respectively in the systems of ordinary differential equations (ODEs), given in equations (1) and (2):

1 $$\begin{array}{rcl}\frac{{\rm{d}}{r}_{1}}{{\rm{d}}t}&=&{R}_{1}-g{r}_{1}{x}_{1}-\alpha g{r}_{1}{x}_{2},\\ \frac{{\rm{d}}{r}_{2}}{{\rm{d}}t}&=&{R}_{2}-\alpha g{r}_{2}{x}_{2},\end{array}$$

2 $$\begin{array}{rcl}\frac{{\rm{d}}{x}_{1}}{{\rm{d}}t}&=&\kappa g{r}_{1}{x}_{1}-{m}_{1}{x}_{1},\\ \frac{{\rm{d}}{x}_{2}}{{\rm{d}}t}&=&\kappa \alpha g({r}_{1}+{r}_{2}){x}_{2}-{m}_{2}{x}_{2},\end{array}$$

where *R*_1_ and *R*_2_ are constants, representing the replenishment rates of resources *r*_1_ and *r*_2_, respectively; *κ* represents the conversion efficiency from resource to offspring, the form of which (for example, as a constant or body-size dependent) does not influence the equilibrium competition outcome (Methods); *m*_1_ and *m*_2_ are the respective mortality rates of consumer species *x*_1_ and *x*_2_. The meaning and units of the parameters are summarized in Supplementary Table 3, and an example of the numerical solution of the system of ODEs is illustrated in Supplementary Fig. 17.

The mortality rate (*m*) is closely associated with body mass and plays a vital role in the competition dynamics between consumers. Here, consumer *i*’s per capita mortality rate is modelled as the product of its home range (*h*_*i*_) and per unit area death rate (*d*_*i*_), both as functions of its body mass (*b*_*i*_). Because body size strongly correlates with many factors, including life history and ecological behaviours^3–5^, competition tends to be stronger between consumers of similar body mass than between those of different body mass^32^, and we focus on analysing the case where the two consumers have the same body mass (*b*_1_ = *b*_2_ = *b*).

3 $${m}_{i}={h}_{i}(b) {d}_{i}(b),\,\,i=1,2.$$

Based on previous findings of animal home range increasing with body mass following a power law^6,14,17,23^, we model home range as

4 $${h}_{i}={b}^{{k}_{i}},$$

where *k*_*i*_ represents the slope of the fitted lines in Supplementary Fig. 18a. We follow Huang et al.^23^ to consider a steeper slope for specialists (that is, with one diet type) than for generalists, thus *k*_1_ > *k*_2_. Furthermore, because larger-sized animals generally have lower external mortality (for example, due to predation) than smaller ones in per unit area, we model the per unit area mortality rate as an S-shaped function of body mass

5 $${d}_{i}=1-\,\mathrm{Exp}\,\left(-\gamma \,\mathrm{Exp}\,(-{b}^{\beta })\right),$$

where *β* and *γ* are parameters that adjust the shape of the function (Supplementary Fig. 18b,c).

**Fig. 5:**
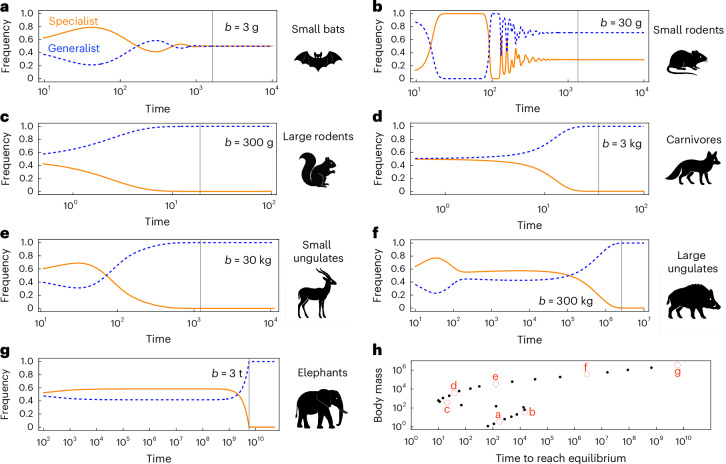
The timescale to reach equilibrium varies with the body masses of competitors. **a**–**g**, Competition dynamics between the specialist (solid orange lines) and the generalist (dashed blue lines) consumer at different body mass (*b* = 3 g, 30 g, 300 g, 3 kg, 30 kg and 3 t). The initial frequency of the specialist was 0.5 across panels. The vertical grey lines indicate the time it takes for the competition dynamics to reach equilibrium. The silhouettes next to each panel illustrate typical mammal subclades at the corresponding range of body mass (see order-specific body size distribution in Supplementary Fig. 3). **h**, Time to reach competition equilibrium varies with body mass, following an S-shaped pattern.

The equilibrium frequency of specialists f^*^ can be derived analytically (Methods):

6 $${f}^{* }=\frac{{b}^{{k}_{2}}{R}_{1}-\alpha {b}^{{k}_{1}}({R}_{1}+{R}_{2})}{{b}^{{k}_{2}}{R}_{1}-\alpha {b}^{{k}_{1}}({R}_{1}+{R}_{2})+{b}^{{k}_{1}}{R}_{2}},$$

which shows that, at equilibrium, the specialist either coexists with the generalist at the frequency f^*^ (Fig. 5a,b) or goes extinct (Fig. 5c–g). The equilibrium frequency of the generalist is 1 − f^*^.

Our series of numerical solutions at different timepoints (Fig. 4 and Supplementary Figs. 12–16) and the analytical results (solid lines in all the figures) show that variations in the parameters can change the body mass ranges where the two consumers can coexist and their frequencies (relative abundances), but do not qualitatively alter the key patterns. Decreasing the steepness of the correlation between body mass and home range for the specialist (decreasing *k*_1_; Fig. 4a) or increasing the steepness of this correlation for the generalist (increasing *k*_2_; Fig. 4b) expands the body mass ranges where the specialist can coexist with the generalist competitor. Reducing the relative resource acquisition rate of the generalist (decreasing *α*; Fig. 4c) or reducing the replenishment rate of the resource accessible only to the generalist (decreasing *R*_2_; Fig. 4f), intuitively, makes the specialist more competitive at all body mass. Adjusting the shape of the per unit area mortality rate function *d* (equation (5), by varying *β* and *γ*) does not influence the competition outcome at equilibrium (equation (6)), but changes the timescale for specialists to maintain coexistence with generalists, especially at large body sizes. For example, a relatively flat *d* function with an early onset of decrease (large *β* and small *γ*; see Supplementary Fig. 18b,c for the effect of varying *β* and *γ*) helps the specialist to coexist with the generalist for a longer time and at higher frequencies (Fig. 4d,e).

The time required for the dynamics to reach equilibrium follows an S-shaped pattern: it first increases and then decreases with competitor body mass, reaching a minimum at around 10^3^ g, and subsequently increases approximately exponentially as body mass continues to grow (Fig. 5). For generality, time is expressed in arbitrary units, primarily to illustrate the relative timescales of the different dynamics and how they approach equilibrium. Between very large animals in particular, although the specialist will eventually be outcompeted by the generalist, they can coexist at relatively stable frequencies for a long time (quasi-stability; Fig. 5f,g). For example, the time it takes for competition between elephant-sized competitors to reach equilibrium is approximately 10^9^ times longer than the time required for competition between squirrel-sized competitors (compare Fig. 5g and Fig. 5c). The large differences in time to reach equilibrium for small- and large-sized competitors, as well as the remarkably long quasi-stability characterizing the competition dynamics between large-bodied animals, suggest that their competition in nature, with frequent biotic and abiotic disturbances, are likely to be far away from equilibrium, adding to the accumulating evidence supporting long transiency as a widespread phenomenon in nature^33^.

Our generic model can be conveniently extended to capture more biological details, such as the competition between specialists and generalists of body-mass-associated dietary features. As an example, we analysed a variation of the model with two resources—a meat-based and a plant-based resource—and three consumers: a meat-eating specialist, a plant-eating specialist and a generalist feeding on both resources (see model extension in the Supplementary Information). The model extension recovers our main result of higher proportions of specialists coexisting with generalists at the smallest and largest extremes of the body mass distribution, showing the robustness of our theory of diverging selection on body size in specialist mammals.

## Discussion

Animal body size has been long recognized as highly relevant to the demand, acquisition and utility of resources^5,11,34^, but the underlying mechanisms are complex and difficult to disentangle. Our combined analyses of empirical data and mathematical models indicated dietary specialization as a key factor influencing how body size diversity is formed and maintained. Across the body size spectrum, we found that the proportion of specialists deviates from the null expectation (defined by the overall proportion) both globally and regionally, contrastingly low in the middle and high towards either or both ends, and this general pattern cannot be explained solely by the phylogenetic signal in body size. These findings suggest that intermediate-sized specialists have less evolutionary advantages than generalists. We built a mathematical model to help demonstrate the advantages of extreme body sizes for specialists, where the largest or smallest specialists are more likely to reach sustainable abundance and coexist with generalists. Therefore, we found both empirical and theoretical support for a diverging selection on the body size of specialists in terrestrial mammals.

Regional faunas can be considered separated natural experiments of macroevolution within their own environmental templates^28,35,36^, for which the zoographic realms (Fig. 2a) are effective units because they contain distinctive assemblages of co-occurring species largely shaped by regional environment and history^28^. Such evolutionary independence is also illustrated by the variation in body size distribution (Supplementary Fig. 7). Neverthless, the patterns of specialist proportion across the range of body size (Fig. 2b) consistently showed that intermediate-sized species within individual regions are more likely to be dietary generalists, as in the global pattern. The absolute position of the lowest proportion varies across regions, potentially reflecting environmental factors in shaping coexistence dynamics, such as how body size scales to mortality (see below). The closest resemblance to the global pattern—where species at both the smallest and largest body sizes are more likely to be specialists than expected—was observed in the Australian and Saharo-Arabian realms. This may result from geographic and environmental isolation, which limits inter-realm dispersals and can complicate evolutionary outcomes by adding or removing taxa either stochastically or under different selective regimes (for example, refs. ^37–40^). The links between body size and specialization in Saharo-Arabia remained detectable in our phylogenetic regression models (Supplementary Table 1), but not in Australia where a few ecologically and evolutionarily distinctive clades coexist, mostly rodents (*n* = 51 species), bats (*n* = 61) and the marsupial species in the orders Dasyuromorphia (*n* = 50) and Diprotodontia (*n* = 60). The marsupials evolved remarkable morphological and functional diversity and resemblance (convergence) to the placental mammals in other continents^41,42^, but their phylogenetic distance from the rodents and bats^43,44^ might have overwhelmed the ecological signal in the body size data for comparative analyses.

Biogeographic effects are also shown from comparisons of other realms, with variation in how specialists are concentrated in extreme-sized species. For example, the proportion of specialists is higher than expected only at the smaller end of the size spectrum in all three New World realms, where Pleistocene megafauna extinctions were most intense^45^. In addition, the Neotropical realm contains a biodiversity hotspot characteristic of closely related lineages^46,47^ and generally smaller body sizes than other continental realms (Supplementary Table 2 and Supplementary Fig. 7). Recent dynamics in the Neotropical landscape, including the Andean uplift and the formation of the Amazon rivers^48^, along with emerging environmental heterogeneity^49^, combined with high productivity and tropical climate (but see below for the Afrotropics), may have favoured smaller-sized specialists over extremely large ones, which require much larger, non-barrier foraging areas^50^. Similarly, small-sized mammals are probably also favoured in the Panamanian realm owing to the narrow land area (Fig. 2a), landscape diversity and, thus, habitat heterogeneity^49,51^. The diversity of mammal body sizes can also be shaped by specific habitats, probably due to the spatial configuration of resources and locomotion requirement, such as fewer intermediate-sized species in open than closed habitats^52^. This habitat effect is not quantifiable at the scale of zoographic realms but could provide mechanistic insights if investigated at a finer spatial resolution.

In comparison with the New World faunas, mammals tend to have larger bodies on the Old World continents (Supplementary Table 2 and Supplementary Fig. 7) but only the Palaearctic and Afrotropical realms show the opposite patterns, where species of the smallest sizes have the regional expected proportion of specialists but those of largest sizes show a higher proportion of specialists than expected (Fig. 2). Patterns in the Sino-Japanese and Oriental realms resemble those in the New World realms. To understand such variation among these connected realms, an integrative approach is needed to compare further factors such as the phylogenetic composition and the biogeographic history of the faunas through landscape evolution and resulting environmental changes^8,11,53^. Notably, in our dataset, only in the two island realms did the proportion of specialists at the largest sizes drop significantly below the regional proportions. In fact, islands represent special cases in terms of both the specialist proportion across size spectrums and the size frequency distribution (Supplementary Fig. 7), possibly because larger-size species (on the global size spectrum, but also large-sized specialists within the realm assemblages) cannot be supported by the limited area and resources in island systems^54,55^. Despite the lack of unanimous support for the ‘island rule’ of body size in current literature, we suggest that it is still a fruitful direction for future research to understand how islands might have different selection regimes with respect to dietary specialization^56,57^, for example, through the incorporation of island biogeographic models (for example, refs. ^58,59^).

When we assessed the variation in body size beyond the phylogenetic effects, we found that large-size species tend to be specialists in regional (defined as realm) assemblages (Supplementary Table 1), suggesting an eco-evolutionary mechanism leading to convergence. On the smaller (left) half of regional size spectrum, a correlation between specialization and body size is often absent in our phylogenetic analyses (except in the Nearctic), suggesting that the higher proportion of dietary specialists at the small end is due to ecological sorting of lineages. The more complex patterns within example orders (Supplementary Fig. 9) also suggest the predominant effects of the external environments on the evolution of body size, so that the co-occurring species need to be considered together in a biogeographic framework. The closest resemblance to the global patterns is in the two richest orders, Rodentia and Chiroptera (bats), which are also two of the few orders that are found in all realms (Supplementary Fig. 8) and representations of small-bodied mammals (Supplementary Fig. 3). The two orders were also found to conform to the global patterns in a previous study that found omnivores more likely to be of intermediate sizes than carnivores and herbivores^60^, though through a comparison using coarser categories of specialization than ours. It is well established that the variation of species’ body size in a clade shows a strong strong phylogenetic signal^27^ (also apparent in our results and Supplementary Fig. 3), even stronger than the spatial structure^26^. When considered from the clade’s perspective, other evolutionary mechanisms might be involved, such as the broadly defined Cope’s rule (the tendency of evolving larger-sized species)^61–63^, which can be further complicated by ecological interactions and environmental factors^8,64,65^. Meanwhile, we suggest further work should consider the sorting mechanisms in forming regional assemblages to explain the diversity in species traits^66,67^. Such ecological sorting might be linked to change in population density and (local) extinctions coinciding with ecological events such as dispersals and environmental changes at regional down to local scales.

Our mathematical model of coexistence provides a theoretical explanation for the nonlinear patterns between body size and dietary specialization shown in the empirical data. General diverging selection on body size towards either gigantism or dwarfism has been found in models of life history evolution^68^, but the links to diet specialization or resource use revealed by our analyses are new insights. Other models have explicitly considered resource types and dietary adaptation but were focused on disruptive selection in sexual dimorphism and adaptive radiation^69–71^. Our model fills in a knowledge gap for understanding animal diversity by explicitly considering dietary specialization and the associated consumption efficiency (*α*; Fig. 4c) in competing consumers of similar body sizes and examining the coexistence outcome across a realistic range of body size.

Based on our model, the reason why coexistence between the specialist and its same-sized generalist competitor is difficult at intermediate body sizes but easier towards the small and large extremes lies in the interactive effects of body size and home range size on mortality risk. These effects can differ in different environmental set-up and ultimately lead to different thresholds of ‘sustainable’ body size in specialists, as suggested by our regional comparisons (Fig. 2). At the (relatively) small extreme of body size, specialists enjoy a reduced mortality risk because they need smaller home ranges to satisfy their resource demand than generalists (Supplementary Fig. 18), owing to a higher efficiency in resource use. At the large extreme of body size, per unit area mortality risk is minimal for both generalists and specialists. In this case, specialists can afford to maintain larger home ranges to allow increased resource intake, if the environment template provides sufficient habitats for them. This assumption of habitat availability should be further explored (for example, varying *R* in Fig. 4f), especially under projections of future environmental changes, because habitat availability has been suggested as a key factor in earlier evolutionary history of terrestrial mammals^11,15,72^. Even with sufficient resources (as in our simplistic model scenario), specialization might still be costly at large bodies and large specialists are expected to eventually go extinct at equilibrium, but the trajectories towards the equilibrium showed that they can coexist with generalists at relatively stable frequencies for a long time (Fig. 5f,g; see also ref. ^62^). Because interspecific competition probably includes long-term metastable transient patterns in most environments^33,73^, we consider the long-term stable coexistence (quasi-stability) between very large specialists and generalists to be more relevant in a realistic context, as it represents a plausible outcome for animals living under fluctuating environmental conditions. We note that incorporating size dependence in the biomass conversion rate (*κ*) does not change the robust pattern of specialists coexisting with generalists in the small and large extremes of body mass in ecologically relevant timescales (Methods; Supplementary Figs. 19 and 20).

Our simple mathematical model serves as a proof of principle demonstrating that ecological differences between dietary specialists and generalists, such as body-size-dependent foraging behaviours and relevant survivorship, can promote the coexistence between specialists and generalists, especially at the small and large extremes of body mass. To further understand the complexity in the empirical patterns, future models should consider (a) the complexity in dietary niche beyond our simple binary variable^74,75^ or the ten categories in our main data source^76^ (Supplementary Fig. 2) and (b) the influence of additional ecological factors on the biomass conversion efficiency (for example, refs. ^6,12,13^), as its variation has been shown to be essential in realistic models of food web adaptive evolution^77^. The vast diversity of plant and animal materials allows much complexity beyond the current data^29,75,78–80^, as, for example, some extreme specialists might be highly selective for the taxon, functional characteristics or growing environment of their food sources. The specific type of diet and, thus, trophic levels are also tightly linked to animals’ foraging behaviours and their metabolic requirements, and empirical evidence has indicated differential selections of body size among mammals of different diets^8,29,60,72,81^. Classic metabolic theories suggest that small bodies, especially in specialists, are better supported by a carnivorous than herbivorous diet, as seen in our data (Fig. 3a), due to the higher mass-specific metabolic rate necessary for compensating their faster heat loss through a larger surface-area-to-volume ratio^4,5,30,31^. When compared among species within the carnivorous or herbivorous group, the deviations from the global patterns (Fig. 3b) could be linked to a common threshold of body size in carnivores related to their prey size (estimated to be 21.5 kg in ref. ^82^; see also ref. ^83^) and, thus, potentially specialization due to energy constraints. Carnivores with a body size above the threshold tend to prey on species having larger bodies than themselves^82^, which could explain the rarity of carnivores towards the larger end of the overall mammalian body size spectrum (Fig. 3; see also ref. ^72^); these large predators tend to be generalists, possibly owing to the generally lower density of larger-sized preys^83,84^. To better understand the body size distribution in different primary dietary groups, especially how such division in prey size (relative to predator size) shapes the body size diversity at lower trophic levels, future models will need to trade off generality in favour of realism and precision^85^ by incorporating group-specific ecological and physiological characters. For example, in ungulate herbivores, the seasonality of their habitat, diet quality and digestive processes (for example, rumination or hindgut fermentation) all influence body size evolution^86^.

Ultimately, we propose that the next steps should expand our model of coexistence ecology to explore macroevolutionary consequences, such as the evolution of the characteristic skewed distribution of mammal body size^87^ (Supplementary Figs. 1, 7 and 10). We must acknowledge that the present-day distribution of biodiversity, including the diversity of body size, arose from diversification (speciations and extinctions) and geographic range dynamics in response to environmental changes at various extents^11,15,62,72,88–90^, including, most recently, multiple glacial–interglacial cycles. Under the influence of environmental change and human activities, the widely known end-Pleistocene extinction preferentially removed large-sized species (the megafauna), including large mammals, at both global and regional scales^91–95^. Investigations through an integrative approach, combining environmental dynamics and biogeography with phylogenetic and fossil data, will be critical for fully understanding the evolutionary mechanisms^66,96^, as well as for anticipating future changes while the resource landscape is continuously modified by anthropogenic factors. However, the nonlinear relationship between body size and specialization calls for caution in using correlation-based analyses, which many macroevolutionary studies rely on. While innovative analytical strategies continue to emerge, simulation models can be a highly effective approach for disentangling complex interactions of biological processes^97,98^ and for interpreting palaeontological data, which are notoriously structured by variation in preservation and sampling (for example, refs. ^99,100^). For example, a useful extension of our model should include how coexistence likelihood varies with the spatial configuration of resources and its dynamics (for example, ref. ^52^), and further help predict the impact of habitat modification. Because the body size and life history of animals can be altered quickly by anthropogenic factors, such as selective harvesting^101^ and habitat loss and fragmentation^102^, those factors and associated eco-evolutionary feedback can form powerful models for predicting future biodiversity.

## Methods

### Empirical investigation of mammal body size and dietary specialization

We compared the body size variation of dietary specialists and generalists globally and within biogeographic regions. Average adult body mass and dietary compositions (density illustrated in Supplementary Fig. 2) for 3,487 terrestrial mammal species were extracted from the EltonTrait database^76^. Body mass of another 877 species in the original dataset was based on a phylogenetic model or the mean at a higher taxonomic level; we excluded these species from our analyses. Based on the resulting dataset (dataset 1), the mammalian species consumed one to six types of dietary material, and we assigned all species with only one diet type as specialists. This simple measure is commonly used in broad-scale comparative analyses^23,74^ and was shown to generate results consistent with other, more complex measures, including the number of diet types and measures that account for the observed clustering patterns among dietary types and the relative consumptions of the different diet types by the same species^23^.

We identified regional assemblages based on the geographic occurrence of terrestrial mammal species across zoographic realms proposed by Holt et al.^28^ (illustrated in Fig. 2a using data downloaded from https://macroecology.ku.dk/resources/wallace on 20 June 2023), accounting for the continuity of regional mammalian assemblages. Biogeographic units such as the zoographic realms are useful in comparative analyses because their boundaries reflect natural divisions of the environmental template, which cause significant turnover in the species composition^28,35,36^. Mammalian species distribution data were downloaded from the International Union for Conservation of Nature Red List database^103,104^ (accessed on 22 June 2023). Species identities were matched to the widely acceptable taxonomy (also used in the EltonTrait database) by Wilson and Reeder^105^, following Fritz et al.^106^, to allow merging with the trait dataset above and the phylogenetic data^43,44^ (see below). The resulting dataset (dataset 1) contains regional occurrences for 3487 species, with the highest species richness in the Neotropical (*n* = 797 species), Oriental (*n* = 771 species) and Afrotropical (*n* = 681 species) faunas (Supplementary Data 1; also summarized in Supplementary Table 2).

In this study, we only included native ranges as defined by the International Union for Conservation of Nature (IUCN), but we acknowledge that human-induced invasions can have non-negligible effects on extinction dynamics and the resulting changes in ecological diversity within regional faunas^107–109^. In the global comparison, we excluded species that only have occurrences assigned to marine habitats or islands by the IUCN^103^ or falling outside the major zoographic realms by Holt et al.^28^. To maximize the comparsion across different geographic regions, we did not exclude the two realms mainly composed of island faunas, Oceanian (296 species) and Madagascan (114 species), but discuss their different patterns above.

The global proportion of specialists in this dataset is 33.4% (out of *n* = 3,487 species). On average, specialists have similar body sizes as generalists (the mean posterior difference and the 5–95% credible interval from a Bayesian regression model (see analytical details below): −0.094 [−0.25 to 0.064]. To illustrate the pattern, we considered the global porportion of specialists as a null hypothesis. We partitioned the data on the basis of every 5% quantile (on average, every 174 species; Supplementary Fig. 1) and, in each of the 20 bins, calculated the proportion of specialist species as well as the 2.5–97.5% binomial proportion confidence interval. Quantiles are used here to generate species pools of similar sample size (number of species), although we acknowledge that some quantiles will cover larger ranges of body size than others due to the uneven distribution of body size frequency (Supplementary Fig. 1). We considered proportions with confidence intervals not containing the global specialist proportion as significant deviations from the null expectation.

Because the variation of body size among mammalian species shows strong phylogenetic signals^27^, we further analysed the data in a multilevel (hierarchical) regression model within a Bayesian framework, incorporating the phylogenetic variance–covariance matrix as a group-level effect (random effect). Coeffecient estimates in such models reflect the potential of ecological associations between the variables^110^. To identify patterns of diverging body sizes for specialists, we estimated the correlation coefficients (coef.) of the number of diet types (fixed effect) with three response variables: (1) the absolute difference from the median body size (*N* = 3,487), (2) body size of species smaller than or equal to the median (*N* = 1,744) and (3) body size of species larger than the median (*N* = 1,743). Body size was logarithm-transformed before the analyses. We used two species-level phylogenetic datasets for mammals with different coverage and estimates of divergence events. The older supertree^44^ (following refs. ^23,106^) contains all species from our trait data and can be directly summarized in one variance–covariance matrix for all regression analyses. We found a significant phylogenetic standard deviation (0.15 [0.15 to 0.16]) in the global model of absolute difference from the median body size, confirming the non-independence of data in the response variable and, thus, the need for phylogenetically informed analyses to investigate ecological and evolutionary associations between focal traits. To assess the robustness of our finding against the uncertainty in phylogenetic reconstruction, we further validated the global model with a more recently published collection of posterior trees^43^, although this dataset misses 75 species from our trait data. We followed the authors’ recommendation and used the trees generated through their node-dating analyses^43^. We respect the 10,000 trees (hosted at https://vertlife.org/data/mammals/, accessed on 31 July 2025) as equally plausible hypotheses and selected 10 random trees to calculate their respective variance–covariance matrices. We then included each matrix in an independent regression model and summarized the posterior estimates of coefficients across the ten models, analysed on the University of Birmingham’s BlueBEAR HPC service. These two sets of phylogenetic data produced consistent results (as did the non-phylogenetic models; Supplementary Table 1), so we base our discussion on the results from the supertree with maximal data coverage. All Bayesian regression models included 5 sampling chains, each with 1,000 iterations after the initial 1,000 warm-up iterations. In all models, we used the default priors and sampling algorithms (Hamiltonian Markov Chain Monte Carlo) provided in the package ‘brms’^111,112^, with a randomization seed of 2,025. Posterior distributions of model coefficients are all summarized on the basis of the mean and the 5–95% quantiles as the credible intervals. Detailed model specifications can be found in the R scripts ‘Analysis_bs_phylogenetics.R’ (using the supertree) and ‘Analysis_bs_phylogenetics_multitree.R’ (using the posterior trees), published with all model outputs.

Based on the same global species pool, we repeated this analysis for regional assemblages in the 11 zoographic realms, each partitioned into 10 quantile bins to ensure sufficient sample sizes for hypothesis testing (Supplementary Fig. 7). We applied the same method (with 10 bins) to comparisons within the five most speciose orders (Supplementary Fig. 10) using the global dataset (excluding species in island realms) to explore the phylogenetic structure of the body size distribution. For analyses on the three primary dietary groups (carnivory, omnivory and herbivory), we excluded species occurring only in the Oceanian and Madagascan realms to focus on continental species, although some island species (for example, the Tasmanian devil in the Australian realm) are still included because they are also found in continental realms (see dataset 1). The remaining data include 931 carnivore species, 1,113 herbivores and 1,335 omnivores. As omnivores are generalists by definition, we analysed the proportion of specialists only for carnivores and herbivores, using 10% body size quantiles and the global proportion within each diet type, respectively. Because, by definition, the number of different specific diet types in carnivores and herbivores is limited, we calculated the proportion of specialists both with and without the addition of omnivores as generalists for comparison (Supplementary Fig. 11). Notably, carnivorous species also tend to have smaller sizes than herbivores as both specialists (one-sided Kolmogorov–Smirnov test: *D* = 0.61, *P* < 0.001) and generalists (*D* = 0.43, *P* < 0.001; Fig. 3a). Among the generalists, omnivores tend to be larger than carnivores (*D* = 0.35, *P* < 0.001; Fig. 3a), but smaller than herbivores (*D* = 0.19, *P* < 0.001; Fig. 3a).

All analyses on the empirical data were conducted in the program R 4.3.1^113^, with the packages ‘tidyverse’^114^ for data organization and illustration, ‘sf’ for spatial analyses^115^, ‘ape’ for processing the phylogenetic data^116^, ‘brms’ for Bayesian multilevel regression analyses^111^, and ‘spData’^117^ and ‘egg’^118^ for data illustration. A dataset ready for analyses is provided as dataset 1. All R code other than the Bayesian regression analyses is provided in ‘Analysis_bs_distributions.R’.

### Derivation and analysis of the equilibrium points

Here, we analytically derive the equilibrium points of the mathematical model given by equations (1) and (2). The equilibrium points are obtained by setting the rates of changes of the variables, that is, the derivatives of the resources and species densities to zero:

7 $$\begin{array}{rcl}0&=&{R}_{1}-g{r}_{1}{x}_{1}-\alpha g{r}_{1}{x}_{2},\\ 0&=&{R}_{2}-\alpha g{r}_{2}{x}_{2},\\ 0&=&\kappa g{r}_{1}{x}_{1}-{m}_{1}{x}_{1},\\ 0&=&\kappa \alpha g({r}_{1}+{r}_{2}){x}_{2}-{m}_{2}{x}_{2}.\end{array}$$

There can be two possible model equilibria. At the first equilibrium, the specialist consumer *x*_1_ goes extinct (*x*_1_ = 0); only the generalist consumer *x*_2_ persists in the system. This gives for the equilibrium densities

8 $$\begin{array}{rcl}{r}_{1}&=&\frac{{R}_{1}{m}_{2}}{\alpha g\kappa ({R}_{1}+{R}_{2})},\\ {r}_{2}&=&\frac{{R}_{2}{m}_{2}}{\alpha g\kappa ({R}_{1}+{R}_{2})},\\ {x}_{2}&=&\frac{\kappa ({R}_{1}+{R}_{2})}{{m}_{2}}.\end{array}$$

At the second equilibrium, the specialist and the generalist consumers coexist. Here, the equilibrium densities of the resources and consumers are

9 $$\begin{array}{rcl}{r}_{1}^{* }&=&\frac{{m}_{1}}{g\kappa },\\ {r}_{2}^{* }&=&\frac{1}{g\kappa }\left(\frac{{m}_{2}}{\alpha }-{m}_{1}\right),\\ {x}_{1}^{* }&=&\frac{\kappa {R}_{1}}{{m}_{1}}-\frac{\kappa {R}_{2}}{\frac{{m}_{2}}{\alpha }-{m}_{1}},\\ {x}_{2}^{* }&=&\frac{\kappa {R}_{2}}{\alpha \left(\frac{{m}_{2}}{\alpha }-{m}_{1}\right)}\end{array}$$

The condition for coexistence requires ${x}_{1}^{* } > 0$, which gives

10 $$\frac{1}{{m}_{1}}{R}_{1}\left(\frac{{m}_{2}}{\alpha }-{m}_{1}\right) > {R}_{2}.$$

Using the above expressions for ${x}_{1}^{* }$ and ${x}_{2}^{* }$, we can find the equilibrium frequency of the specialist density

11 $${f}^{* }=\frac{{x}_{1}^{* }}{{x}_{1}^{* }+{x}_{2}^{* }}=\frac{\frac{1}{{m}_{1}}{R}_{1}\left(\frac{{m}_{2}}{\alpha }-{m}_{1}\right)-{R}_{2}}{\frac{1}{{m}_{1}}{R}_{1}\left(\frac{{m}_{2}}{\alpha }-{m}_{1}\right)+\left(\frac{1}{\alpha }-1\right){R}_{2}}.$$

Inserting the expressions of *m*_1_ and *m*_2_ in equation (3) into equation (11), we obtain the equilibrium frequency of the specialist consumer in equation (6).

The conversion efficiency from resource to offspring (*κ*) does not affect the equilibrium competition outcome either (equation (6)). Intuitively, this is because we focus on the competition between consumers of the same body mass; the effects of body mass on *κ* are the same and, thus, cancel out. Still, different forms of *κ* can influence transient competition dynamics leading to the equilibrium. To illustrate this, we implemented an alternative form of *κ* following Brown et al.^6^:

12 $$\kappa (b)=\frac{{C}_{0}{b}^{{b}_{0}}{C}_{1}{b}^{{b}_{1}}}{{C}_{0}{b}^{{b}_{0}}+{C}_{1}{b}^{{b}_{1}}},$$

where *b*_0_, *b*_1_, *C*_0_ and *C*_1_ are constants, with parameter values taken from ref. ^6^. The frequencies of the specialist competitor produced with *κ* following equation (12) and with *κ* as a constant are different during transient competition dynamics (compare Supplementary Figs. 13 and 19), while they are very similar at quasi-stability (compare Fig. 4 and Supplementary Fig. 20). This suggests that body-mass-dependent conversion efficiency from food to offspring may play a role in short-term ecological interaction dynamics but does not change whether the competitors can coexist in the long term.

To determine the stability of the equilibrium state, we numerically checked the eigenvalues of the corresponding Jacobian matrices, in which we substituted the equilibrium densities in equation (9). For all model parameters, regardless of whether both consumers coexist or only the generalist consumer persists, we found the coexistence equilibrium point to be locally stable. Numerical solutions of the competition dynamics and equilibrium points were performed using the software Mathematica. The notebook file is provided in ‘MathematicaNotebook.nb’, which also contain the analysis of our model extension described in the ‘Appendix’ in the Supplementary Information.

### Reporting summary

Further information on research design is available in the Nature Portfolio Reporting Summary linked to this article.

## Supplementary information

Supplementary Information Appendix and Supplementary Tables 1–3, Figs. 1–20 and References.

Reporting Summary

Supplementary Data 1 A summary dataset for the empirical analyses.

## Data Availability

No new data have been generated in this study, and all analyses are based on data from public databases. A synthesis dataset ready for analyses and generating the figures is provided as dataset 1. In addition, all model output from Bayesian regression analyses is available via Figshare at 10.6084/m9.figshare.27303813 (ref. ^119^).

## Code availability

The R code for the empirical analyses, including data illustration and the Mathematica code for generating numerical solutions, is available via Figshare at 10.6084/m9.figshare.27303813 (ref. ^119^).
