# Supplementary material for: Diverging selection on body size in specialist terrestrial mammals
Source: Nat Ecol Evol. 2026 Jan 28;10(2):342–54. doi: 10.1038/s41559-025-02959-2 (PMC12890589; doi:10.1038/s41559-025-02959-2)
Supplement: Supplementary file 1 — Appendix and Supplementary Tables 1–3, Figs. 1–20 and References. [file 41559_2025_2959_MOESM1_ESM.pdf]

nature ecology & evolution

Supplementary information

https://doi.org/10.1038/s41559-025-02959-2

Diverging selection on body size in specialist terrestrial mammals

In the format provided by the authors and unedited

Contents

1 Appendix: Extended mathematical model **2**

1.1 A model considering body mass-related dietary features of the competitors 2

1.2 Analysis of the equilibrium states of the supplementary model 9

2 Supplementary Tables **15**

3 Supplementary Figures **16**

4 Supplementary References **35**

1 Appendix: Extended mathematical model

1.1 A model considering body mass-related dietary features of the competitors

The modelling framework presented in the main results is general and can be readily extended to incorporate more biological specificities relevant to specific research systems. Here, we present an example that considers a system of two resources (i.e., r_P, a plant-based resource, and r_A, an animal-based resource), a specialist consumer x_P, which only feeds on the plant-based resource, a specialist consumer x_A, which only feeds on the animal-based resource, and a generalist consumer x_G, which consumes both resources. The specialists consume their corresponding resources at rate g, while the generalist consumes resources less efficiently than the specialists, at a rate αg for both resources, with $0 < \alpha < 1$. The temporal dynamics of the resources and the abundance of the three consumers can be summarized in the following system of ordinary differential equations eq. (A1) and eq. (A2), respectively.

$$\begin{aligned} \frac{dr_P}{dt} &= R_P - gr_Px_P - \alpha gr_Px_G, \\ \frac{dr_A}{dt} &= R_A - gr_Ax_A - \alpha gr_Ax_G \end{aligned} \tag{A1}$$

$$\begin{aligned} \frac{dx_P}{dt} &= \kappa gr_Px_P - m_Px_P, \\ \frac{dx_A}{dt} &= \kappa gr_Ax_A - m_Ax_A, \\ \frac{dx_G}{dt} &= \kappa\alpha g(r_A + r_P)x_G - m_Gx_G. \end{aligned} \tag{A2}$$

In eq. (A1), R_P and R_A are constants, representing the replenishment rates of the plant- and animal-based resources, respectively, and the replenishment rate of the plant-based resource is s times of the animal-based resource; In eq. (A2), κ is a constant, representing the conversion rate from food to offspring (see the implementation of an alternative, body mass-dependent form of κ below); m_P, m_A, and m_G are the mortality rates of the plant-eating specialist, the animal-eating specialist, and the generalist, respectively.

The mortality rate (m) is closely associated with body mass and plays a vital role in the competition dynamics between consumers. Specifically, we model the mortality rate of a species as the product of its home range size (h) and per unit area mortality rate (d):

$$m_i = h_i \cdot d_i, \quad i = P, A, G, \tag{A3}$$

The home ranges of the consumers scale with their body mass (b) and are influenced by the proportions of plant-based resource ($0 \leqslant p_{P_i} \leqslant 1$) and the animal-based resource ($p_{A_i} = 1 - p_{P_i}$) in their diet, thus $p_{P_G} = r_P/(r_A + r_P)$ for the generalist.

$$\begin{aligned} h_i &= b^{k_i}, \\ k_i &= k_0 + k_{VO_{2\max}} \cdot p_{P_i}^{\delta_P} + k_{\text{BMR}} \cdot p_{A_i}^{\delta_A}. \quad i = P, A, G, \end{aligned} \tag{A4}$$

The exponent k_i is influenced by the proportion of plant-based resource in the diet through the maximum metabolic rate ($VO_{2\max}$), which is the rate of oxygen consumption during the maximum sustainable rate of exercise (e.g., during chasing for prey or escaping from a predator). A large proportion of plant-based food in the diet contributes to a larger contribution of the component of $k_{VO_{2\max}}$ to the scaling exponent k_i. Intuitively, this reflects the fact that a cheetah needs to outrun the slowest springbok to catch a meal, while a springbok needs to outrun the fastest cheetah to survive. The relative contribution of the component $k_{VO_{2\max}}$ to k_i as a whole is adjusted by the proportion of plant-based resource (p_P) in the diet by the parameter δ_P, which determines the shape of the dependence relationship curve: when $\delta_P = 1$, the contribution of $k_{VO_{2\max}}$ increases linearly with p_P; when $0 < \delta_P < 1$, the contribution of $k_{VO_{2\max}}$ concaves downwards as p_P increases; when $\delta_P > 1$, the contribution of $k_{VO_{2\max}}$ concaves upwards.

The exponent k_i in eq. (A4) is also influenced by the proportion of animal-based resource in the diet through the basal metabolic rate (BMR), which has been shown to increase with the proportion of meat in the diet in carnivores for which nature selection favours muscle fibers with a high mitochondria density that enhance endurance rather than power [1]. We model the positive influence of meat-eating on BMR by scaling k_{BMR} with p_A, the proportion of animal-based resource in the diet. The parameter δ_A, similar to δ_P, adjusts the convavity of the dependence.

Because smaller animals are more likely to die from extrinsic causes (e.g. predation) than larger animals in per unit area of their home range, we model the per unit area mortality rate of animals as an S-shaped decreasing function of body mass,

$$d_i = 1 - \text{Exp}\left(-\gamma \text{Exp}(-b^{\beta})\right), \tag{A5}$$

where β and γ are parameters that adjust the shape of the function. The S-shaped monotonically decreasing function has a range between 0 and 1, capturing the empirical observation that the per unit area mortality rate due to extrinsic causes generally decreases with body size. In other words, very small animals experience a high mortality rate, but very large animals seldom die from external causes.

There are four distinct states at the quilibrium of the ecological interactions: (i) two specialists coexist while the generalist goes extinct; (ii) the generalist coexist with the specialist that consumes the more abundant resource; (iii) the generalist outcompetes both specialists; and (iv) all three consumers coexist, which only occurs under specific parameter combinations. See fig. A1 and fig. A2 for illustrations of the ecological dynamics leading to each equilibrium state. The analyses of the equilibrium states are provided in SI section 1.2.

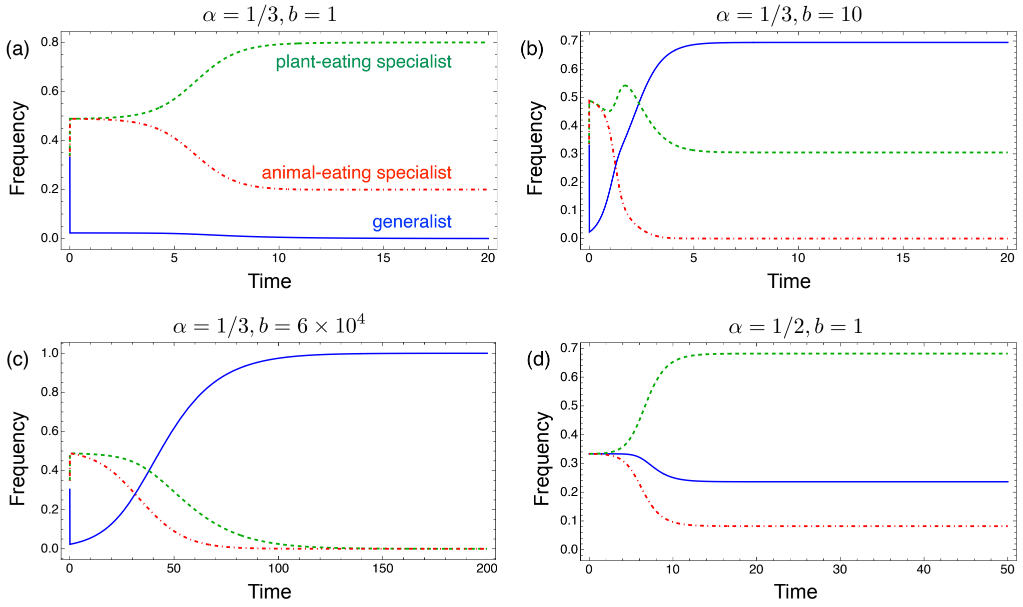

Fig. A1 Four possible equilibrium states of the competition outcome: (a) The two specialists coexist while the generalist goes extinct at the equilibrium; (b) The generalist and one of the specialists (here the plant-eater) coexist; (c) Only the generalist persist; and (d) All three consumers coexist. Line colors and types are consistent across panels. Here the conversion rate of resources into offspring was set to a constant, $\kappa = 10$. The other parameter values were set to $\alpha = 1/3$, $\beta = 0.25$, $\gamma = 100$, $\delta_P = \delta_A = 3$, $g = 10$, $k_0 = k_{VO_{2\max}} = k_{\mathrm{BMR}} = 0.4$, $R_A = 1$, $s = 4$.

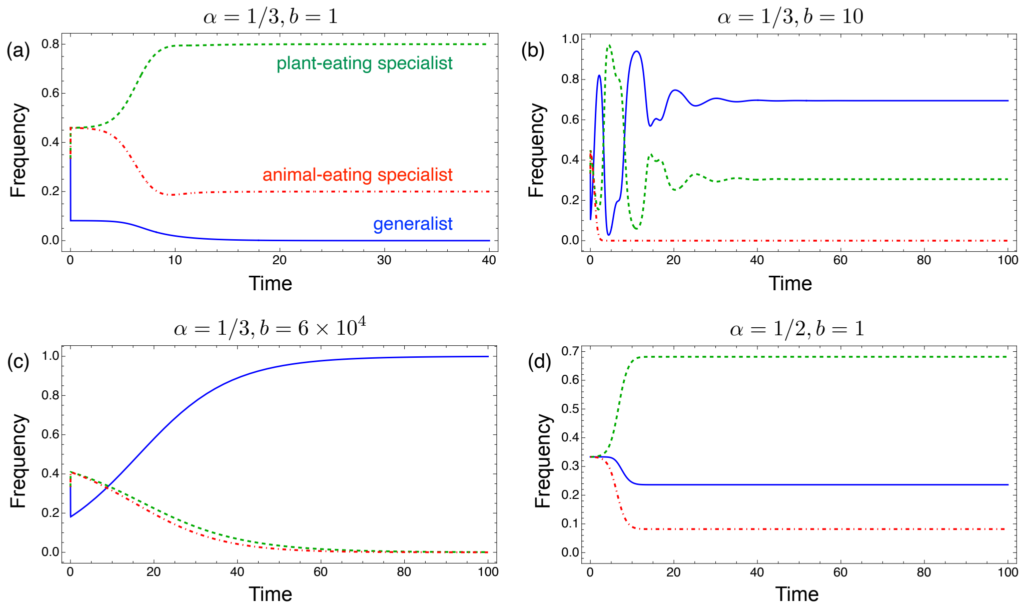

Fig. A2 Four possible equilibrium states of the competition outcome. The only difference from fig. A1 is that the conversion rate from food to offspring depends on body mass, following Eq. (**??**). The additional parameter values are $b_0 = 0.75$, $b_1 = -0.25$, $C_0 = 6$, $C_1 = 0.2$.

The time it takes for the system to reach equilibrium generally increases with body mass. Among very large animals, although the specialist will eventually become outcompeted by the generalist, they can coexist at high frequencies for a very long time (e.g., more than 10^7 generations for animals at a body mass of 10^6 g). The competition trajectories in fig. A3 (κ set to constant) and fig. A4 (κ follows eq. (12) of the main text) show the time scale it takes for the system to reach equilibrium at different body mass.

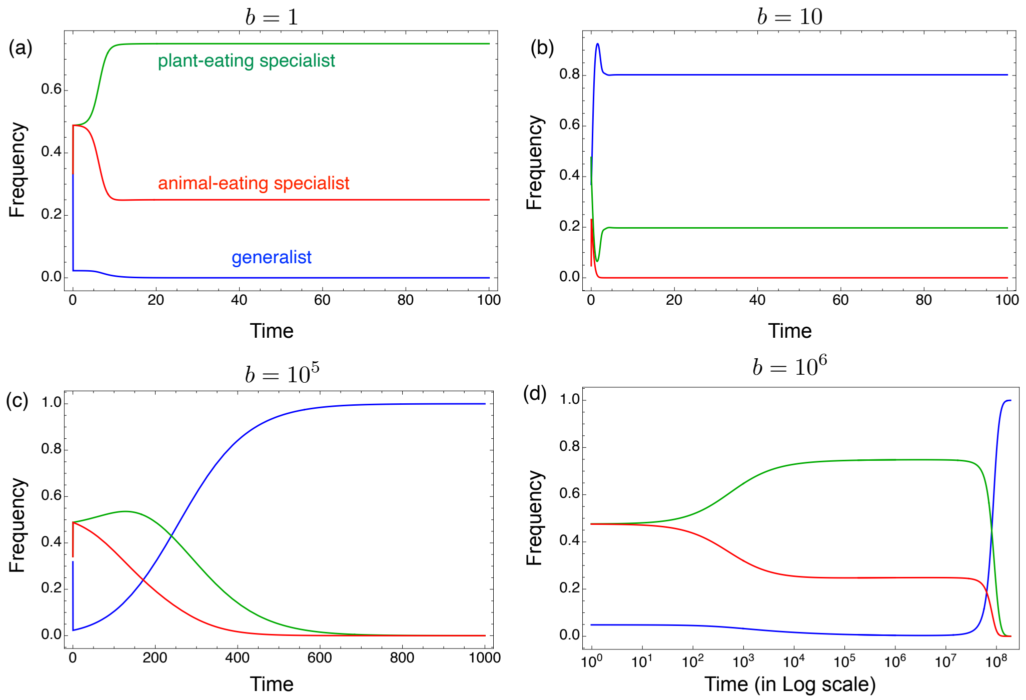

Fig. A3 Competition dynamics between the specialist and generalist consumers. The time it takes for the system to reach equilibrium generally increases with body mass. Note the x-axis in panel (d) is in Log scale. Line colors and types are consistent across panels. Here the conversion rate from food to offspring is set to a constant, $\kappa = 10$. The other parameter values are $\alpha = 1/3$, $\beta = 0.25$, $\gamma = 100$, , $g = 1$, $k_0 = k_{VO_{2\max}} = k_{\mathrm{BMR}} = 0.4$, $R_A = 1$, $s = 3$.

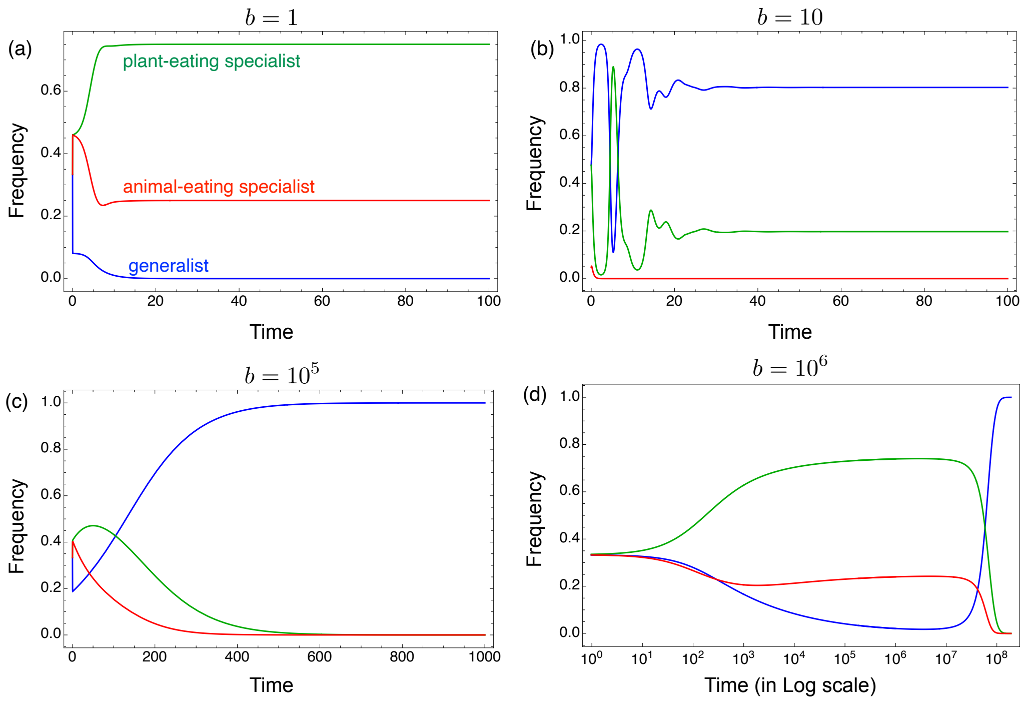

Fig. A4 Competition dynamics between the specialist and generalist consumers. The only difference from fig. A3 is that the conversion rate from food to offspring depends on body mass, following eq. (12) of the main text. The additional parameter values are $b_0 = 0.75$, $b_1 = -0.25$, $C_0 = 6$, $C_1 = 0.2$.

For comparison purposes, we produced two sets of numerical solutions for the total frequency of the specialists at arbitrarily selected time points allowing quasi-stability after initial oscillations (at $t = 1000$ in fig. A5 and $t = 10000$ in fig. A6. Our results show that increasing the resource acquisition rate of the generalist relative to the specialists (α), intuitively, makes the specialists less competitive (fig. A5a, fig. A6a). The competitiveness of the specialists can be promoted, at large body masses by increasing the shape parameter β or decreasing γ (fig. A5b-c, fig. A6b-c), and at small to intermediate body masses by decreasing δ_P and δ_A (fig. A5d-e, fig. A6d-e). Finally, a biased resource replenishment rate (s) promotes the persistence of specialists at small or large body masses.

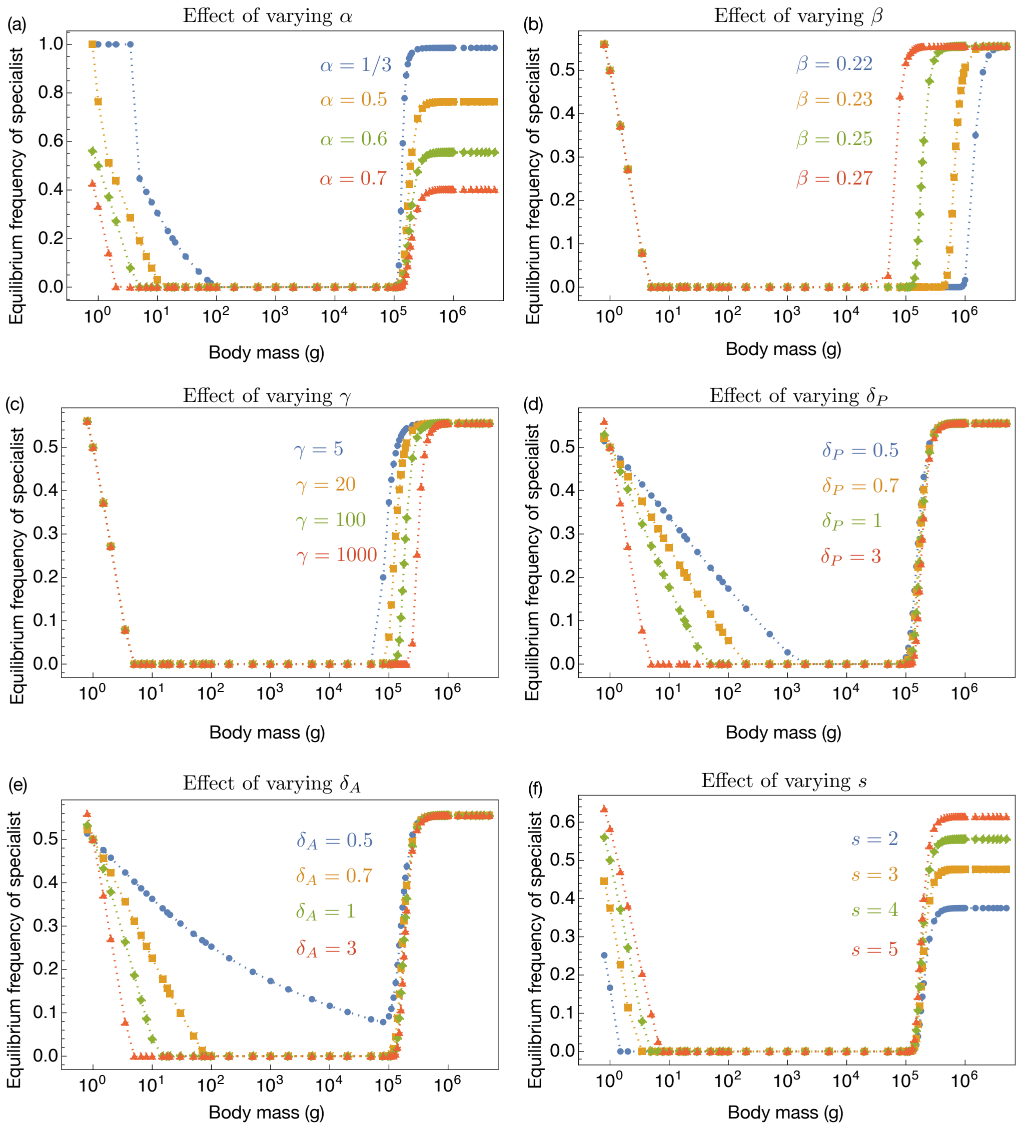

Fig. A5 Total frequency of the specialist consumers at quasi-stability. The conversion rate from food to offspring was set to a constant, $\kappa = 10$. The data points are generated numerically at $t = 1000$. The other parameter values are: $g = 10$, $R_A = 1$, $k_0 = k_{VO_{2\max}} = k_{\text{BMR}} = 0.4$.

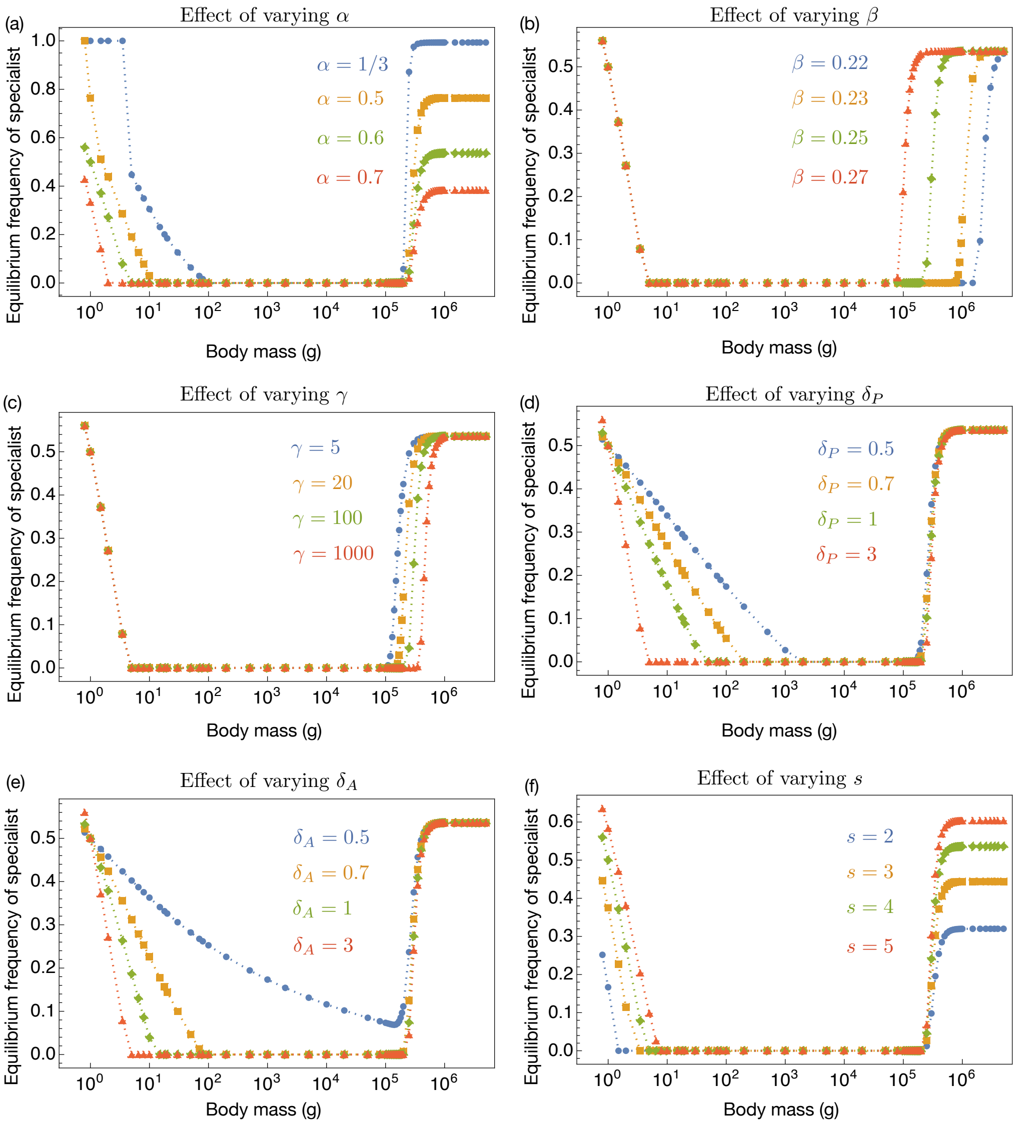

Fig. A6 Total frequency of the specialist consumers at quasi-stability. The conversion rate from food to offspring was set to a constant, $\kappa = 10$. The data points were generated numerically at $t = 10000$. The other parameter values are: $g = 10$, $R_A = 1$, $k_0 = k_{VO_{2\max}} = k_{\mathrm{BMR}} = 0.4$.

Furthermore, to show that the conversion rate from food to offspring (κ) does not significantly influence the frequency of specialists at quasi-stability, we implemented an alternative form of κ following eq. (12) of the main text and numerically calculated the total frequency of the specialists at quasi-stability ($t = 10000$). The results in fig. A7 is quantitively similar to those in fig. A5 and fig. A6.

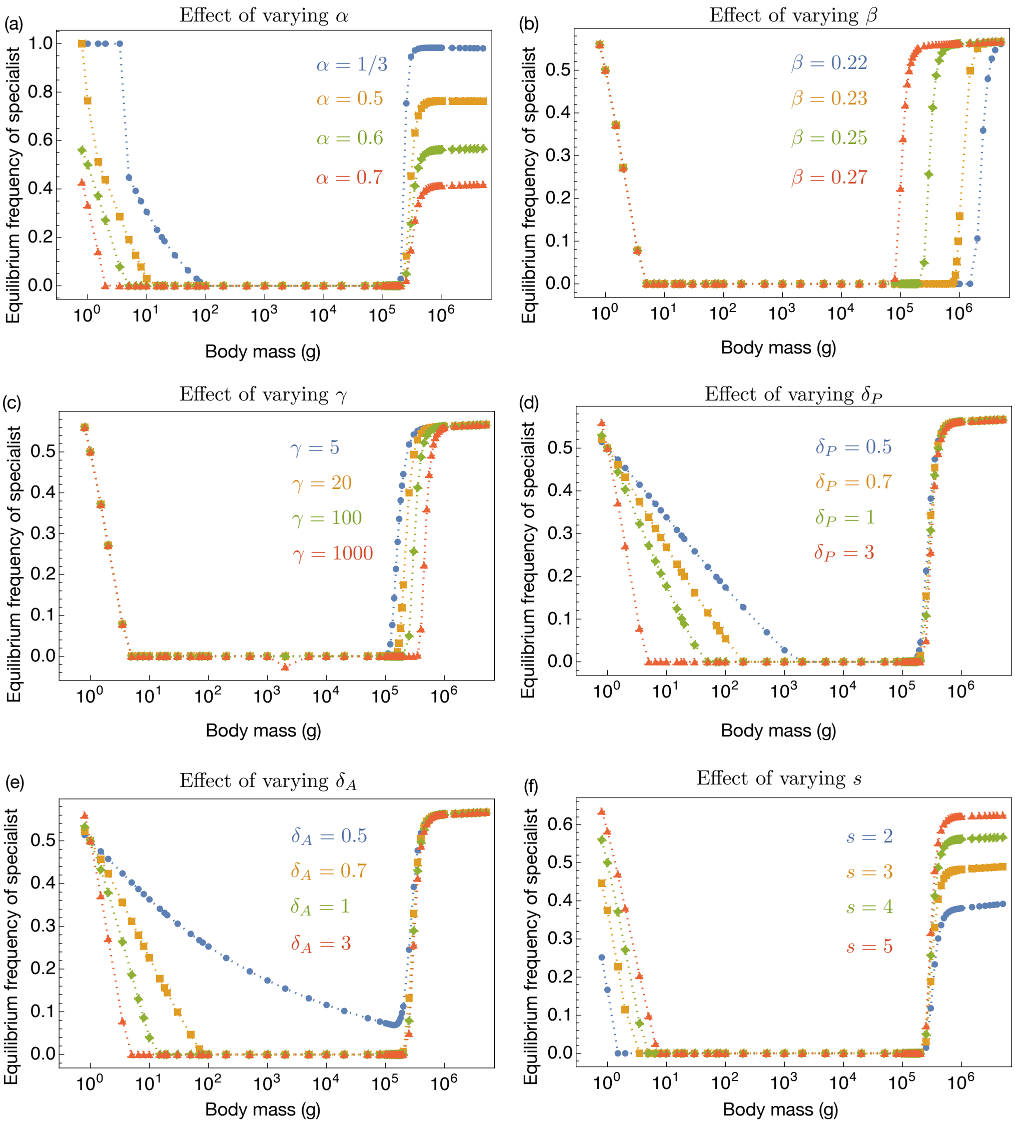

Fig. A7 Total frequency of the specialist consumers at quasi-stability. The conversion rate from food to offspring was implemented following eq. (12) of the main text. The data points were generated numerically at $t = 10000$. The other parameter values are: $b_0 = 0.75$, $b_1 = -0.25$, $C_0 = 6$, $C_1 = 0.2$, $g = 10$, $R_A = 1$, $k_0 = k_{VO_{2\max}} = k_{\mathrm{BMR}} = 0.4$.

1.2 Analysis of the equilibrium states of the supplementary model

Here we analytically derive the equilibrium points of the mathematical model described by eq. (A1) and eq. (A2). The stationary states (equilibrium points) are determined by setting the time

derivatives to zero. We thus have the following system of algebraic equations:

$$\begin{aligned}
0 &= R_P - g r_P x_P - \alpha g r_P x_G, \\
0 &= R_A - g r_A x_A - \alpha g r_A x_G, \\
0 &= \kappa g r_P - m_P, \\
0 &= \kappa g r_A - m_A, \\
0 &= \kappa \alpha g (r_A + r_P) - b^{f\left(\frac{r_P}{r_P + r_A}\right)} d,
\end{aligned} \tag{A6}$$

where the function f denotes

$$f\left(\frac{r_P}{r_P + r_A}\right) = k_0 + k_{VO_{2\max}} \cdot \left(\frac{r_P}{r_P + r_A}\right)^{\delta_P} + k_{\mathrm{BMR}} \cdot \left(1 - \frac{r_P}{r_P + r_A}\right)^{\delta_A}. \tag{A7}$$

The coexistence of all three consumers in an equilibrium state is possible only under the special case when $\alpha = 1/2$ and $b = 1$ (fig. A1d and fig. A2d). Otherwise, two species can coexist at maximum. Indeed, from the third and fourth equations of eq. (A6) we have the stationary values for r_P and r_A,

$$\begin{aligned}
r_P &= \frac{m_P}{\kappa g}, \\
r_A &= \frac{m_A}{\kappa g}.
\end{aligned} \tag{A8}$$

Substituting eq. (A8) into the fifth equation of eq. (A6), we obtain

$$0 = \alpha (m_P + m_A) - b^{f\left(\frac{m_P}{m_A + m_P}\right)} d. \tag{A9}$$

For an arbitrary (non-degenerate) set of parameter values, the above identity would be wrong. Therefore, the system of equations for the stationary states for all three consumers is unsolvable, if we aim to find positive values of all consumer densities. Therefore, we further consider the three generally possible scenarios, where the density of at least one consumer type is zero.

(i) The stationary densities of both specialist consumers are zeros, and only the generalist consumer remains. The stationary state in this case is given by $(r_P^*, r_A^*, 0, 0, x_G^*)$. The corresponding

equations for r_P^*, r_A^*, x_G^* read

$$\begin{aligned}
0 &= R_P - \alpha g r_P^* x_G^*, \\
0 &= R_A - \alpha g r_A^* x_G^*, \\
0 &= \kappa \alpha g (r_A^* + r_P^*) - b^{f\left(\frac{r_P^*}{r_P^* + r_A^*}\right)} d.
\end{aligned} \tag{A10}$$

From the first and second equations of eq. (A10) we have

$$\begin{aligned}
r_P^* &= \frac{R_P}{\alpha g x_G^*}, \\
r_A^* &= \frac{R_A}{\alpha g x_G^*}.
\end{aligned} \tag{A11}$$

We substitute eq. (A11) into the third equation of eq. (A10) to obtain

$$\kappa (R_P + R_A) = x_G^* b^{f\left(\frac{r_P}{r_P + r_A}\right)} d. \tag{A12}$$

Then we get the solution as

$$\begin{aligned}
r_P^* &= \frac{R_P}{\alpha g x_G^*}, \\
r_A^* &= \frac{R_A}{\alpha g x_G^*}, \\
x_G^* &= \frac{\kappa (R_P + R_A)}{b^{f\left(\frac{r_P}{r_P + r_A}\right)} d}.
\end{aligned} \tag{A13}$$

The stability of the state $(r_P^*, r_A^*, 0, 0, x_G^*)$ is determined by the necessary conditions

$$\begin{aligned}
\kappa g r_P^* - m_P &< 0, \\
\kappa g r_A^* - m_A &< 0.
\end{aligned} \tag{A14}$$

This condition follows from the requirement that x_A and x_P should not invade when their initial densities are small

$$\begin{aligned}
\frac{dx_P}{dt} &= \kappa g r_P x_P - m_P x_P < 0, \\
\frac{dx_A}{dt} &= \kappa g r_A x_A - m_A x_A < 0.
\end{aligned} \tag{A15}$$

We should stress that the relative frequency of the specialists will be zero for this given state.

(ii) The stationary density of the generalist consumer is zero, and only specialist consumers remain. The stationary state is given by $(r_P^{**}, r_A^{**}, x_P^{**}, x_A^{**}, 0)$. The corresponding equations for (r_P^{**},r_A^{**}, x_P^{**}, and x_A^{**} are given by

$$
\begin{aligned}
0 &= R_P - g r_P^{**} x_P^{**}, \\
0 &= R_A - g r_A^{**} x_A^{**}, \\
0 &= \kappa g r_P^{**} - m_P, \\
0 &= \kappa g r_A^{**} - m_A.
\end{aligned} \tag{A16}
$$

This gives the stationary densities as

$$
\begin{aligned}
r_P^{**} &= \frac{m_P}{\kappa g}, \\
r_A^{**} &= \frac{m_A}{\kappa g}, \\
x_P^{**} &= \frac{\kappa R_P}{m_P}, \\
x_A^{**} &= \frac{\kappa R_A}{m_A}.
\end{aligned} \tag{A17}
$$

The necessary condition for the stability of this state is

$$
\alpha(m_P + m_A) - b^{f\left(\frac{r_P}{r_P + r_A}\right)} d < 0. \tag{A18}
$$

In this case, the rate of change of x_G around the equilibrium should fulfill the condition

$$
\frac{dx_G}{dt} = \kappa \alpha g (r_A + r_P) x_G - b^{f\left(\frac{r_P}{r_P + r_A}\right)} d x_G < 0. \tag{A19}
$$

Therefore, under this condition, x_G will die out when it is rare. Clearly, the frequency of the specialists (in the absence of the generalist) will be one.

(iii) The stationary density of one specialist species is zero, whereas that of the other one is positive. Also, the generalist is present in the system. For simplicity, we may consider that the density of the meat-eating specialist is zero ($x_A = 0$). For the case where $x_P = 0$, the calculations will be similar. The state is denoted by $(r_P^{***}, r_A^{***}, x_P^{***}, 0, x_G^{***})$. The stationary state equations

read as

$$
\begin{aligned}
0 &= R_P - g r_P^{***} x_P^{***} - \alpha g r_P^{***} x_G^{***}, \\
0 &= R_A - \alpha g r_A^{***} x_G^{***}, \\
0 &= \kappa g r_P^{***} - m_P. \\
0 &= \kappa \alpha g (r_A^{***} + r_P^{***}) - b^{f\left(\frac{r_P^{***}}{r_P^{***}+r_A^{***}}\right)} d.
\end{aligned}
\tag{A20}
$$

From the third and fourth equations of eq. (A20), we have

$$
\begin{aligned}
r_P^{***} &= \frac{m_P}{\kappa g}, \\
\kappa \alpha g \frac{r_A^{***} + r_P^{***}}{r_P^{***}} r_P^{***} &= b^{f\left(\frac{r_P^{***}}{r_P^{***}+r_A^{***}}\right)} d.
\end{aligned}
\tag{A21}
$$

Denote $\frac{r_A^{***}+r_P^{***}}{r_P^{***}} = p^\dagger$, we obtain the equation for $p^\dagger$,

$$
p^\dagger m_P \alpha = b^{f(1/p^\dagger)} d. \tag{A22}
$$

This is a transcendental equation for $p^\dagger$, which can be solved only numerically.

Now, let us suggest that we know the solution of $p^\dagger$ of the equation. Then we can find r_A^{***} as

$$
r_A^{***} = r_P^{***}(p^\dagger - 1) = \frac{m_P}{\kappa g}(p^\dagger - 1). \tag{A23}
$$

We can then derive the stationary densities of species. From the second line of eq. (A20), we have

$$
x_G^{***} = \frac{\kappa R_A}{\alpha m_P (p^\dagger - 1)}. \tag{A24}
$$

From the first line of eq. (A20), we have

$$
x_P^{***} = \frac{R_P(p^\dagger - 1) - R_A}{\frac{m_P}{\kappa}(p^\dagger - 1)}. \tag{A25}
$$

The necessary condition of stability for the given stationary state is valid under the condition that

$$
\kappa g r_A^{***} - m_A < 0. \tag{A26}
$$

In this case

$$\frac{dx_A}{dt} = \kappa g r_A x_A - m_A x_A < 0, \tag{A27}$$

meaning that the density of the meat-eating specialist consumer should decrease to zero when it is initially rare. Therefore, the total frequency of specialists will be given by $\frac{x_P^{***}}{x_P^{***}+x_G^{***}}$, where the stationary densities are defined above. In a similar way, one can derive the expressions for the stationary density in the case where the plant-eating specialist goes extinct.

A crucial aspect of the above stationary states is the verification of their stability. Note that the above-introduced conditions based on checking the possibility of invasion of the absent species, when rare, only provide necessary conditions of stability. Therefore, one needs to formally apply standard linear analysis using the Jacobian matrix computed at the stationary state, which results in cumbersome expressions. Therefore, we numerically checked the eigenvalues of the corresponding 5x5 Jacobian matrices, in which we substituted the above-mentioned equilibrium densities. For all model parameters, we found that the above necessary conditions based on the invasion of the absent species become sufficient for local stability of the equilibrium points. We do not show here the corresponding expressions for the Jacobian matrices for the sake of brevity. All numerical solutions of the competition dynamics and equilibrium points have been performed using the software Mathematica.

2 Supplementary Tables

Realm	combined coefficient	left coefficient	right coefficient
Neotropical	-0.0054 [-0.067, 0.055]	0.038 [-0.029, 0.1]	0.017 [-0.074, 0.11]
Oriental	0.004 [-0.062, 0.069]	0.035 [-0.066, 0.14]	-0.024 [-0.098, 0.054]
Afrotropical	-0.053 [-0.12, 0.014]	-0.049 [-0.15, 0.048]	**−0.11 [-0.2, -0.016]**
Panamanian	0.036 [-0.051, 0.12]	0.057 [-0.035, 0.15]	0.058 [-0.063, 0.18]
Nearctic	**-0.16 [-0.25, -0.08]**	**0.23 [0.12, 0.33]**	**-0.16 [-0.28, -0.043]**
Palearctic	**-0.18 [-0.25, -0.1]**	0.11 [-0.0051, 0.23]	**-0.2 [-0.3, -0.11]**
Sino-Japanese	**-0.11 [-0.2, -0.015]**	0.091 [-0.05, 0.23]	**-0.12 [-0.23, -0.0044]**
Saharo-Arabian	**-0.1 [-0.18, -0.021]**	0.06 [-0.1, 0.23]	**-0.13 [-0.23, -0.033]**
Oceanian	**-0.15 [-0.27, -0.034]**	0.095 [-0.06, 0.25]	-0.15 [-0.32, 0.013]
Australian	-0.029 [-0.16, 0.11]	-0.0019 [-0.16, 0.16]	-0.074 [-0.28, 0.13]
Madagascan	0.0062 [-0.12, 0.13]	-0.12 [-0.38, 0.15]	-0.026 [-0.15, 0.09]
**Global**	**-0.044 [-0.074, -0.014]**	**0.049 [0.0087, 0.09]**	**-0.042 [-0.082, -0.0019]**

Table S1 Coefficient estimates (with 5-95% credible intervals from the posterior distribution) for the number of diet types in predicting body size globally (bottom row) and by zoographic realms, accounting for phylogenetic effect in a multi-level regression model. To identify the non-linear relationship, estimates were based on the absolute difference from the median body size (combined coefficient) as well as separately for the sizes smaller (left coefficient) and larger (right coefficient) than the median. The number of diet type ranges from 1 to 6, the body size data (mass, in g) was logarithm-transferred, and a species-level phylogeny [2, following 3, 4] was converted to a variance-covariance matrix to be incorporated in the model. Strong phylogenetic effect on body size is indicated by the phylogenetic sd in the global model (0.15 $[0.15, 0.16]$). We repeated the global analyse using 10 randomly selected trees from a more recently published collection of posterior phylogenetic datasets which contain 75 fewer species [5], and found a consistent negative correlation between the number of diet type and distance from median body size: (coef. $= -0.034$ $[-0.065, -0.0026]$, with phylogenetic sd $= 0.18$ $[0.17, 0.19]$), which is also qualitatively consistent with our non-phylogenetic regression analysis (coef. $= -0.22$ $[-0.26, -0.18]$).

Zoographic realm	Number of species	Mean body size (mass in g)	Standard devitiation of size	Proportion of dietary specialist (%)
Sino-Japanese	375	31842	187730	43.5
Afrotropical	681	30891	211274	34.7
Palearctic	444	25037	108112	36.5
Oriental	771	20083	150780	37.1
Nearctic	454	14049	72816	32.4
Saharo-Arabian	297	13151	41559	42.1
Australian	239	13054	112355	39.7
Neotropical	797	6173	61597	28.9
Panamanian	464	3570	20855	31.5
Oceanian*	296	1165	6101	47.6
Madagascan*	114	1057	1742	46.5
**Global**	**3487**	**14685**	**125154**	**33.4**

Table S2 Summary statistics of the 11 regional assemblages defined by the zoographic realms proposed by Holt et al.[6], with the global summaries in the last row. *Realms mainly composed of island faunas.

Parameter	Meaning	Unit
R_1, R_2	Replenishment rates of resource r_1 and r_2, respectively	$[r][t]^{-1}$
g	per-capita resource consumption coefficient	$(\text{individual} \cdot [t])^{-1}$
α	consumption-rate ratio of x_2 relative to x_1	Dimensionless
κ	conversion efficiency from consumed resource to offspring	$\text{individual} \cdot [r]^{-1}$
m_1, m_2	mortality rate of x_1 and x_2, respectively	$[t]^{-1}$

Table S3 Meaning and units of parameters in systems of differential equations (1) and (2) of the main text. $[r]$ represents the unit of resource, e.g., g or kg; $[t]$ represents the unit of time, e.g., month, year or decade.

3 Supplementary Figures

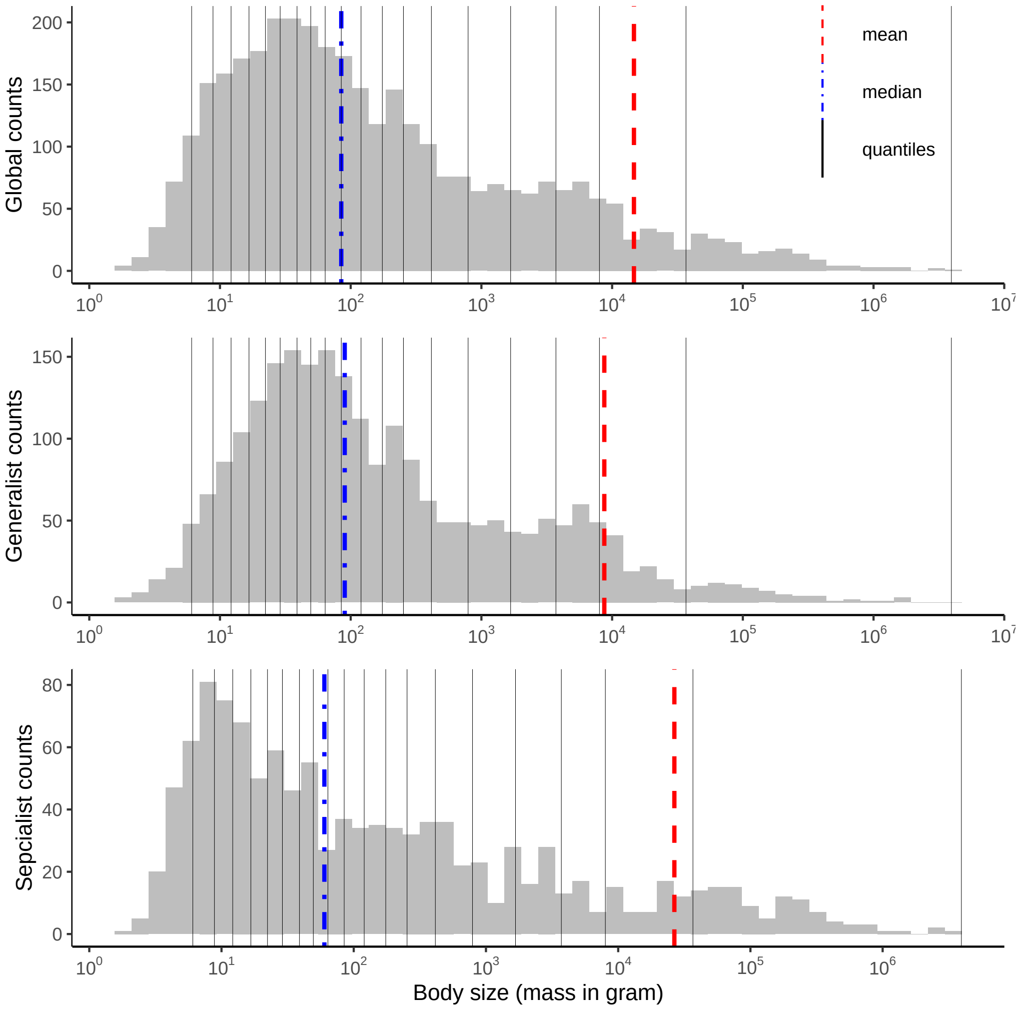

Fig. S1 Frequency distribution of body size in terrestrial mammals in our global analysis ($n = 3495$). The black solid vertical lines show our partition of the data for calculating the proportion of specialists. The red dashed and blue dotted-dashed line indicate the mean and median respectively.

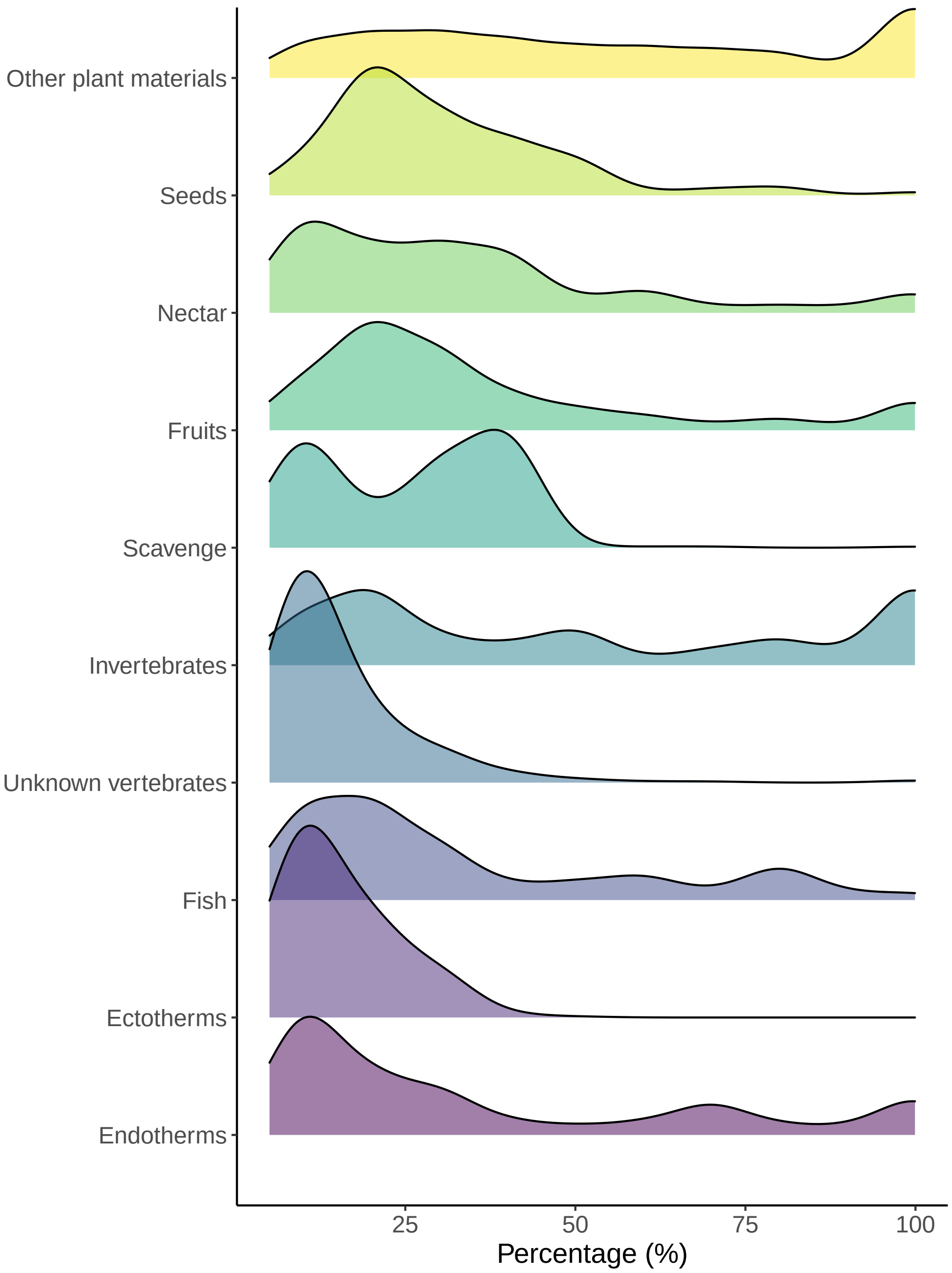

Fig. S2 Density distribution of diet composition in terrestrial mammals from the EltonTrait database [7]. Illustration was based on 3487 species included in our analyses.

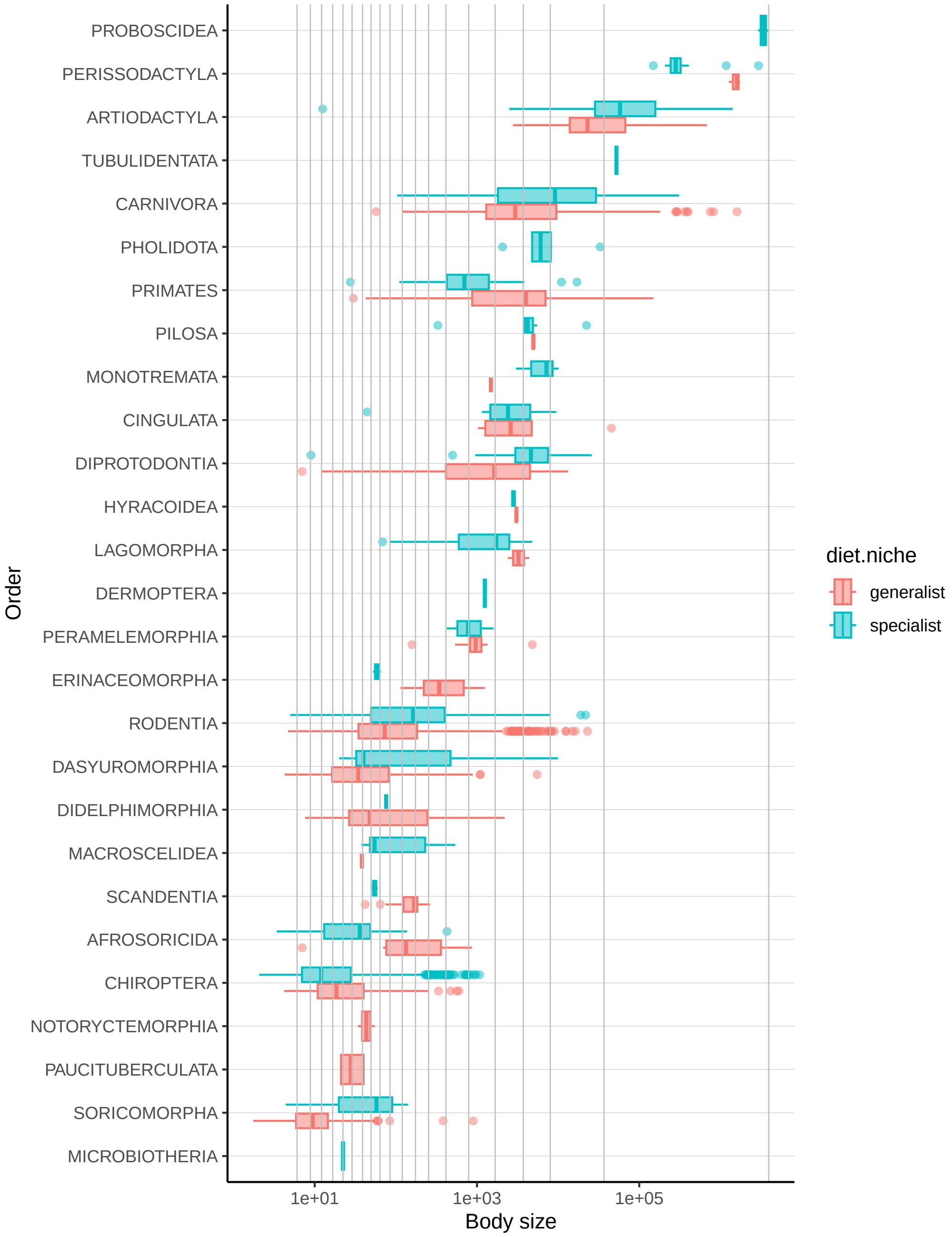

Fig. S3 The body size distributions vary across the 28 taxonomic orders reflecting influences by their shared evolutionary history and intrinsic biological properties. The vertical grey lines indicate the 20 (5 − 95%) quantiles used in our analyses (e.g. illustrated in Fig.1 of the main text and fig. S1).

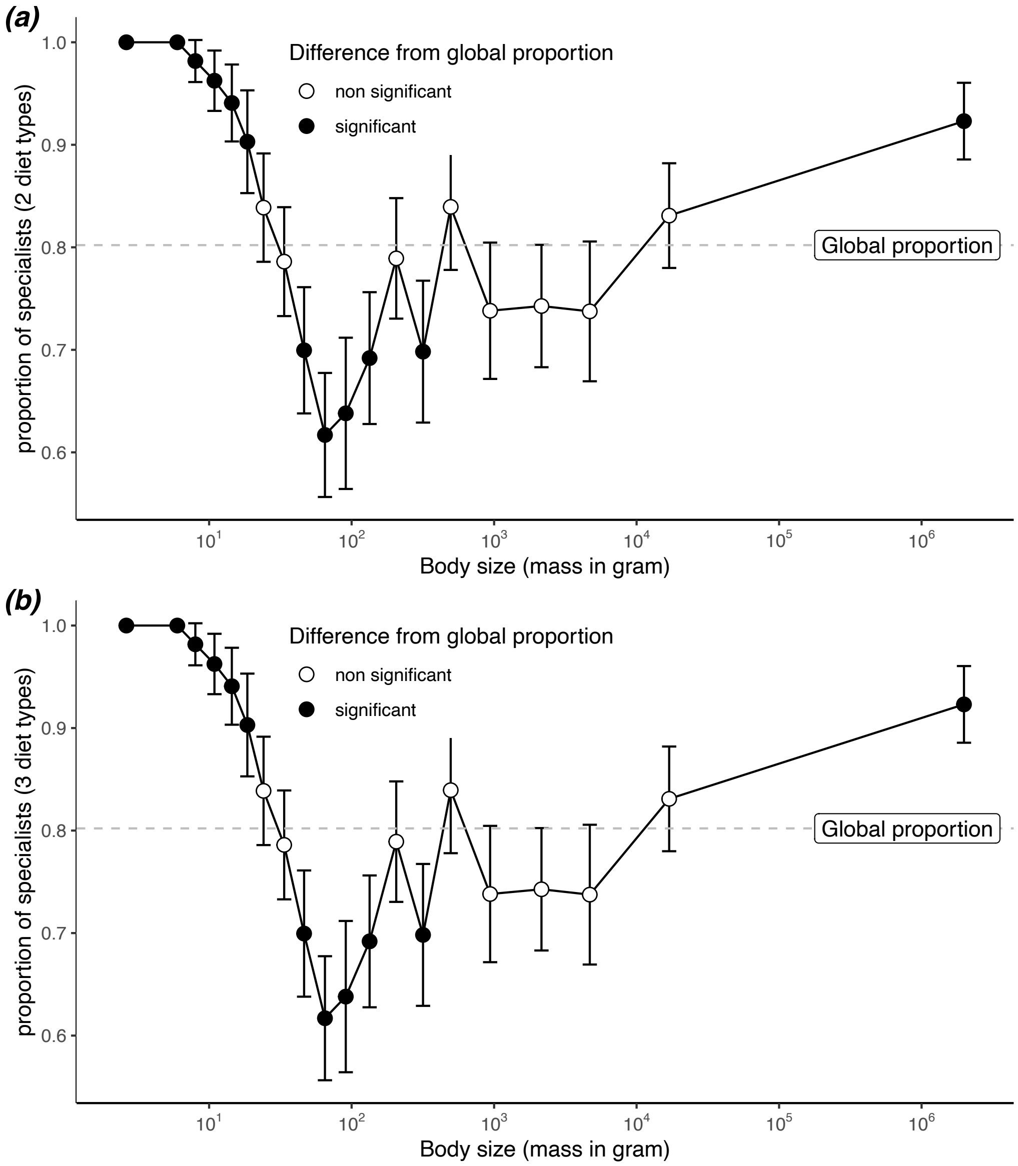

Fig. S4 Consistent patterns with Fig. 1 of the main text when different cutoffs were used to separate specialists and generalists. The proportion of dietary specialists (points) is generally high in species with small and large body sizes but low in species with intermediate sizes (compared in 20 bins of species partitioned by each 5% quantiles of average adult body mass). The error bars represent the 95% binomial proportion confidence intervals. Proportions were onsidered significant deviations from the null expetation (solid points) when their confidence intervals do not contain the proportion of specialists in all mammal species (indicated by the gray dashed line).

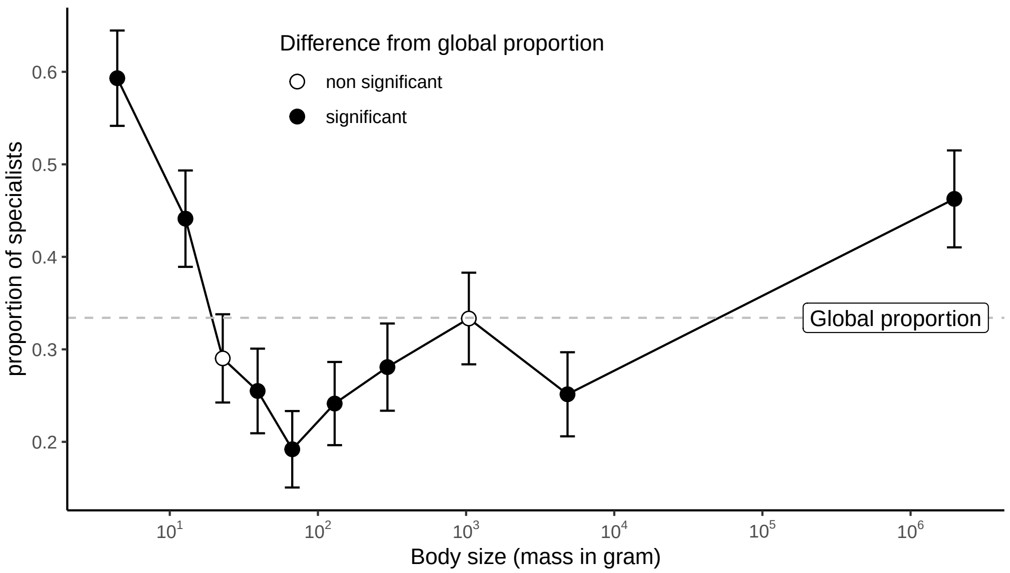

Fig. S5 Consistent patterns with Fig. 1 of the main text when analyzed using 10 bins (partitioned by each 10% quantiles of average adult body mass) to provide a direct comparison with the regional patterns in Fig. 2 of the main text: the proportion of dietary specialists (points) is generally low in species with intermediate sizes but high at extreme sizes. The error bars represent the 95% binomial proportion confidence intervals. Proportions were onsidered significant deviations from the null expetation (solid points) when their confidence intervals do not contain the proportion of specialists in all mammal species (indicated by the gray dashed line).

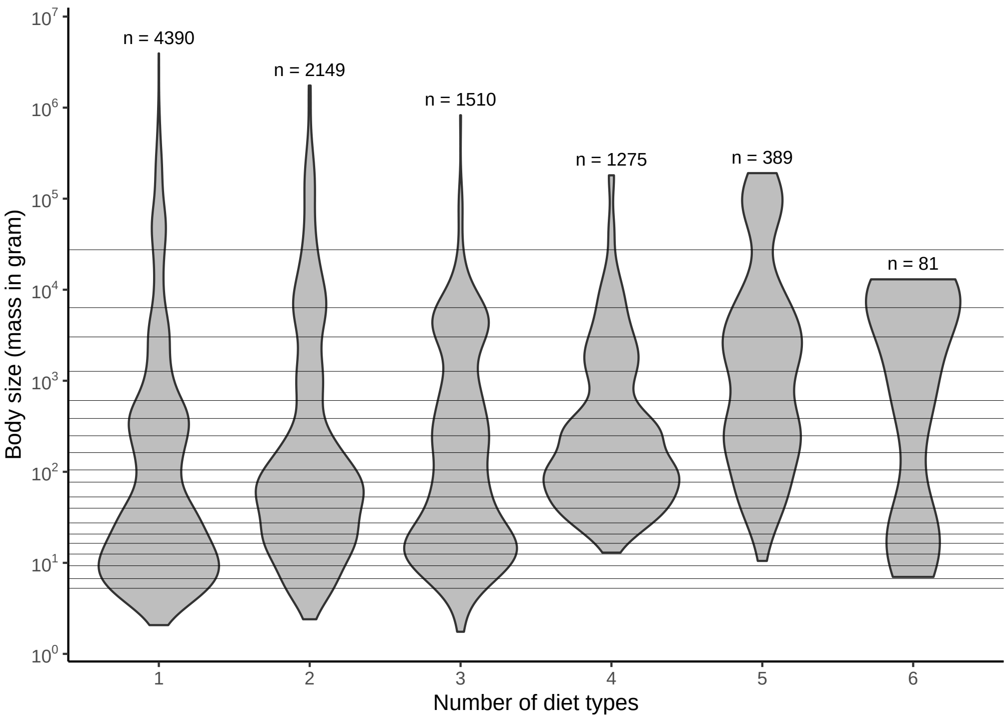

Fig. S6 The body size distributions vary across a range of dietary specialisation, with species consuming more diet types, thus less specialized, clustered around intermediate sizes. The horizontal lines indicate the 20 $(5 - 95\%)$ quantiles used in our analyses (e.g. illustrated in Fig.1 of the main text and fig. S1). The number of species in each group is given above the density distribution represted by the violins.

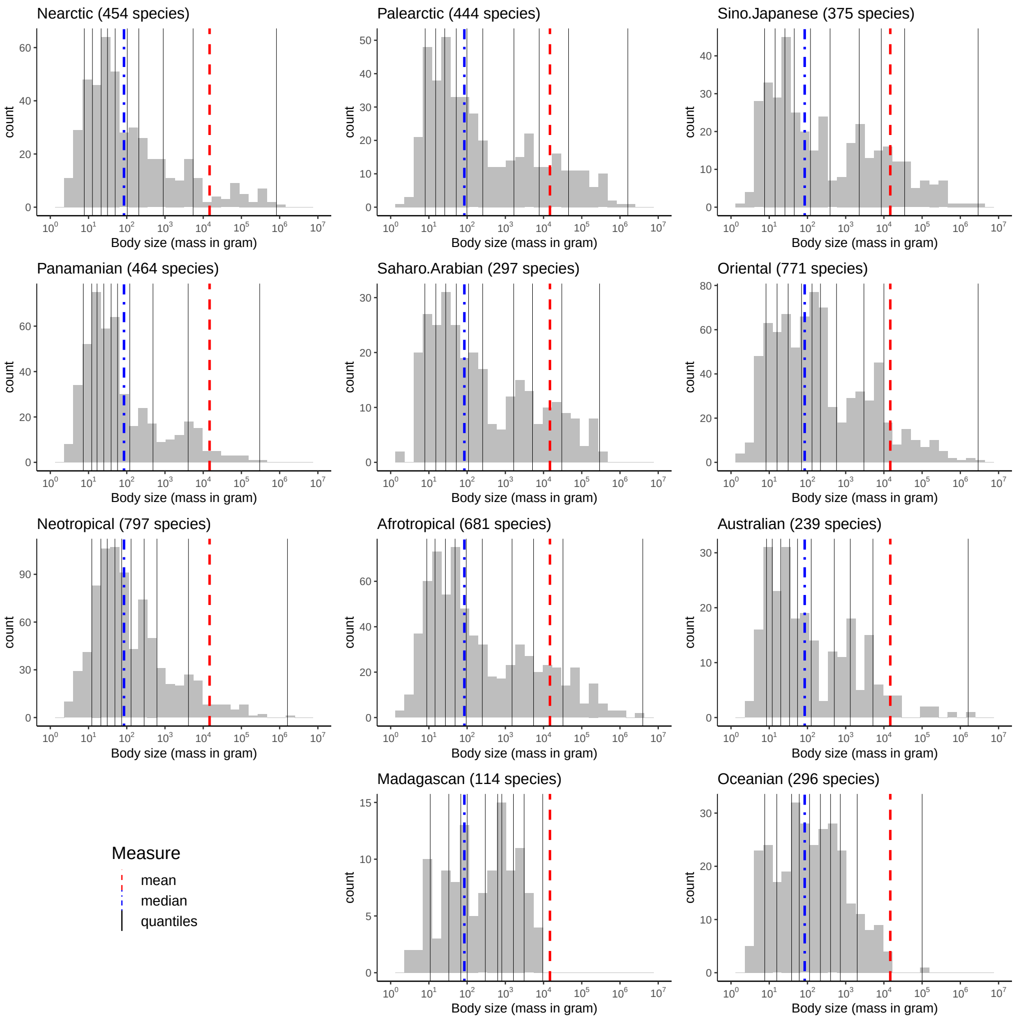

Fig. S7 Frequency distribution of body size in terrestrial mammals in our regional analyses, based on the zoographic realms identified by Holt et al. [6], as illustrated in Fig 2A of the main text. The black solid vertical lines show our partition of the data for calculating the proportion of specialists in each realm, where the total sample sizes are indicated in the panel headings. The red dashed and blue dotted-dashed lines indicate the means and medians respectively.

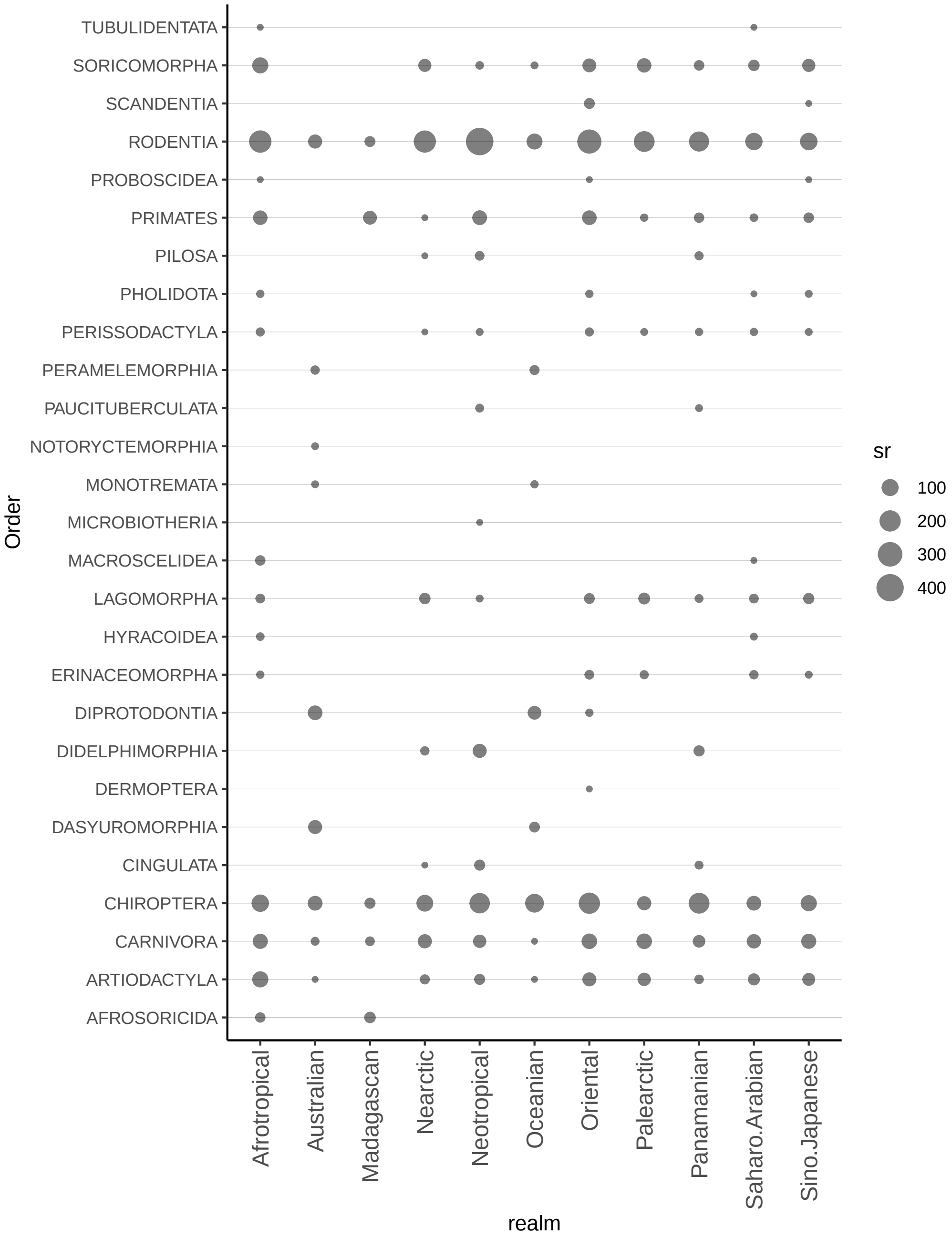

Fig. S8 Most orders have species in multiple realms and are thus potentially subjected differential selections by regional environments and mammalian assemblages

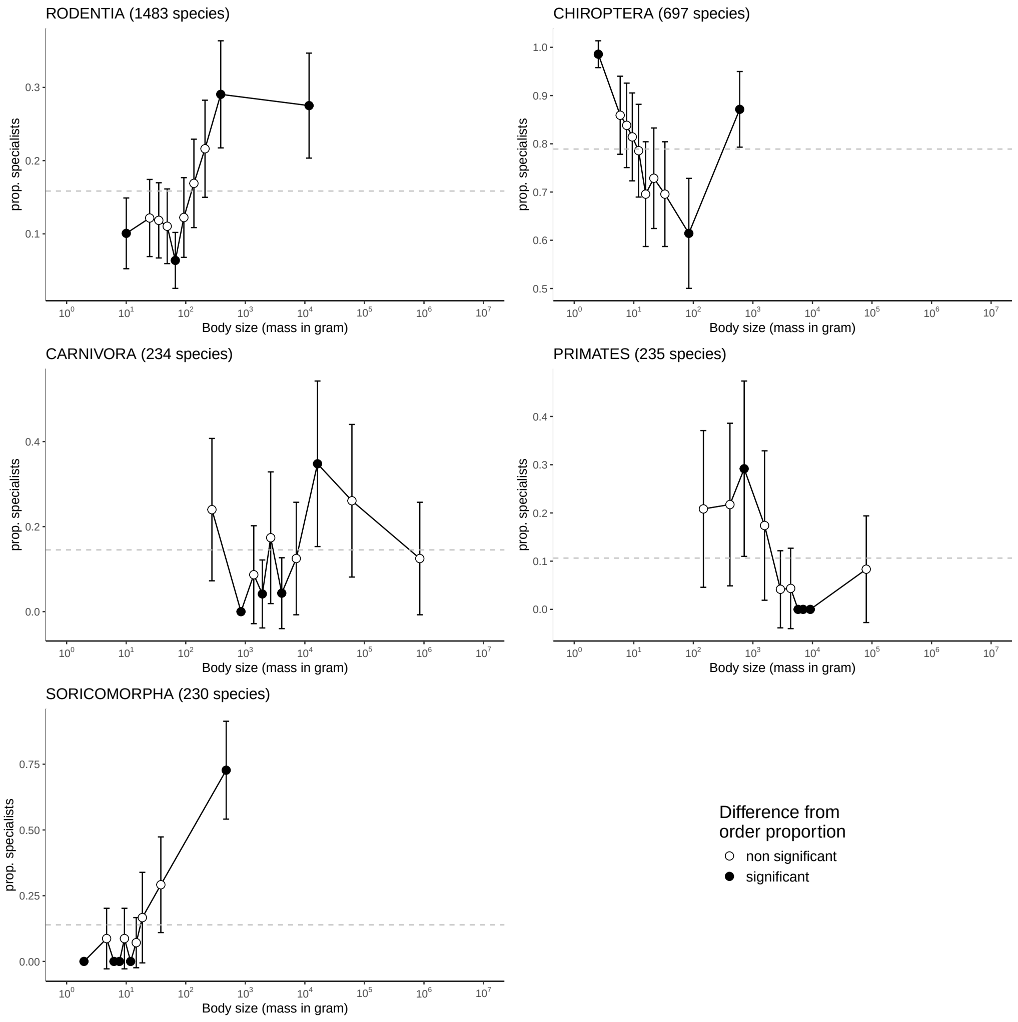

Fig. S9 In the five most species-rich orders (richness indicated in the panel titles), the proportion of dietary specialists (points) drops in species with non-extreme sizes (compared in 10 bins of species partitioned by each 10% quantiles of average adult body mass, see Figure S12). We considered proportions whose 95% binomial proportion confidence intervals (error bars) do not contain the proportion of specialists in the order (indicated by the gray dashed line) as significant deviations from the null expectation (solid points).

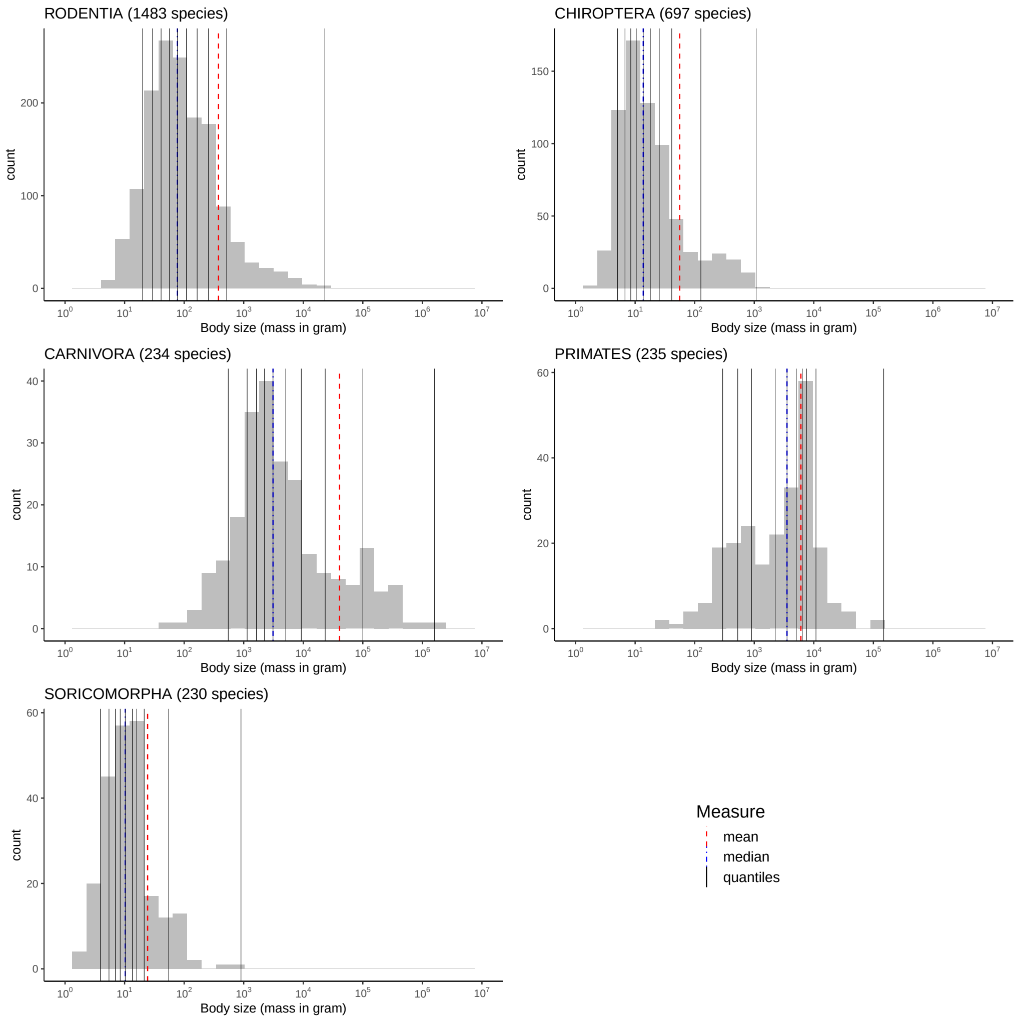

Fig. S10 Frequency distribution of body size in the five taxonomic orders with most species (richness indicated in the panel titles). The black solid vertical lines show our partition of the data for calculating the proportion of specialists in each order. The red dashed and blue dotted-dashed lines indicate the means and medians respectively.

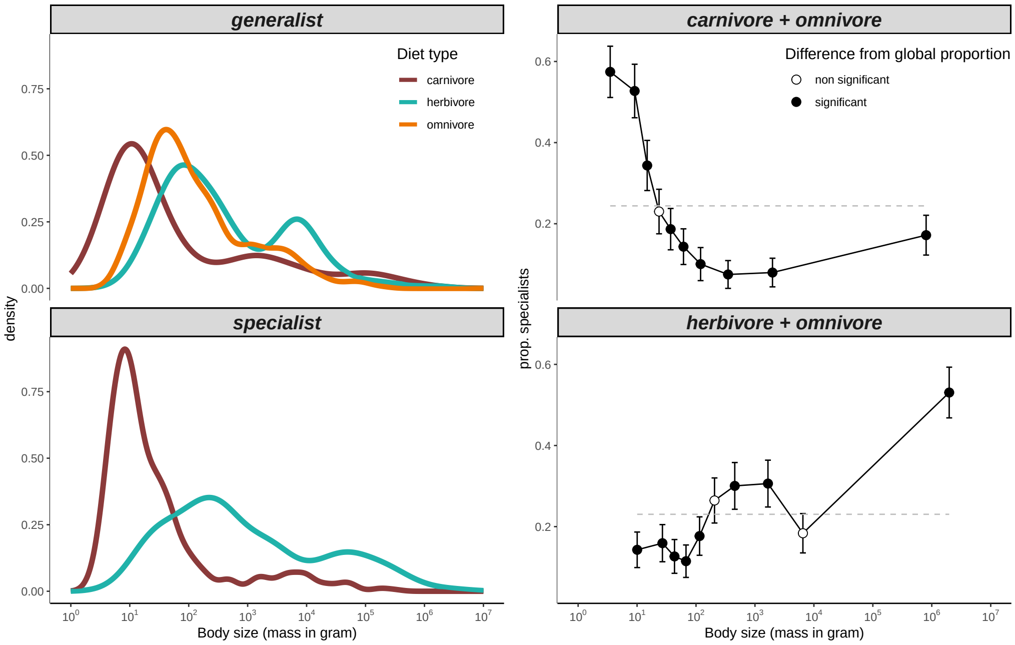

Fig. S11 Because omnivores tend to be larger than generalist carnivores ($D = 0.45$, $p < 0.001$, Fig. 3a of the main text), the global proportion of animal-eating specialists is much lower than the proportion at smallest sizes when we consider omnivores as generalists in the group. In contrast, omnivores tend to be smaller than generalist herbivores ($D = 0.19$, $p < 0.001$, Fig. 3a of the main text) so that adding omnivores lowers both the global proportion and the small-sized proportions of specialists further for plant-eating mammals.

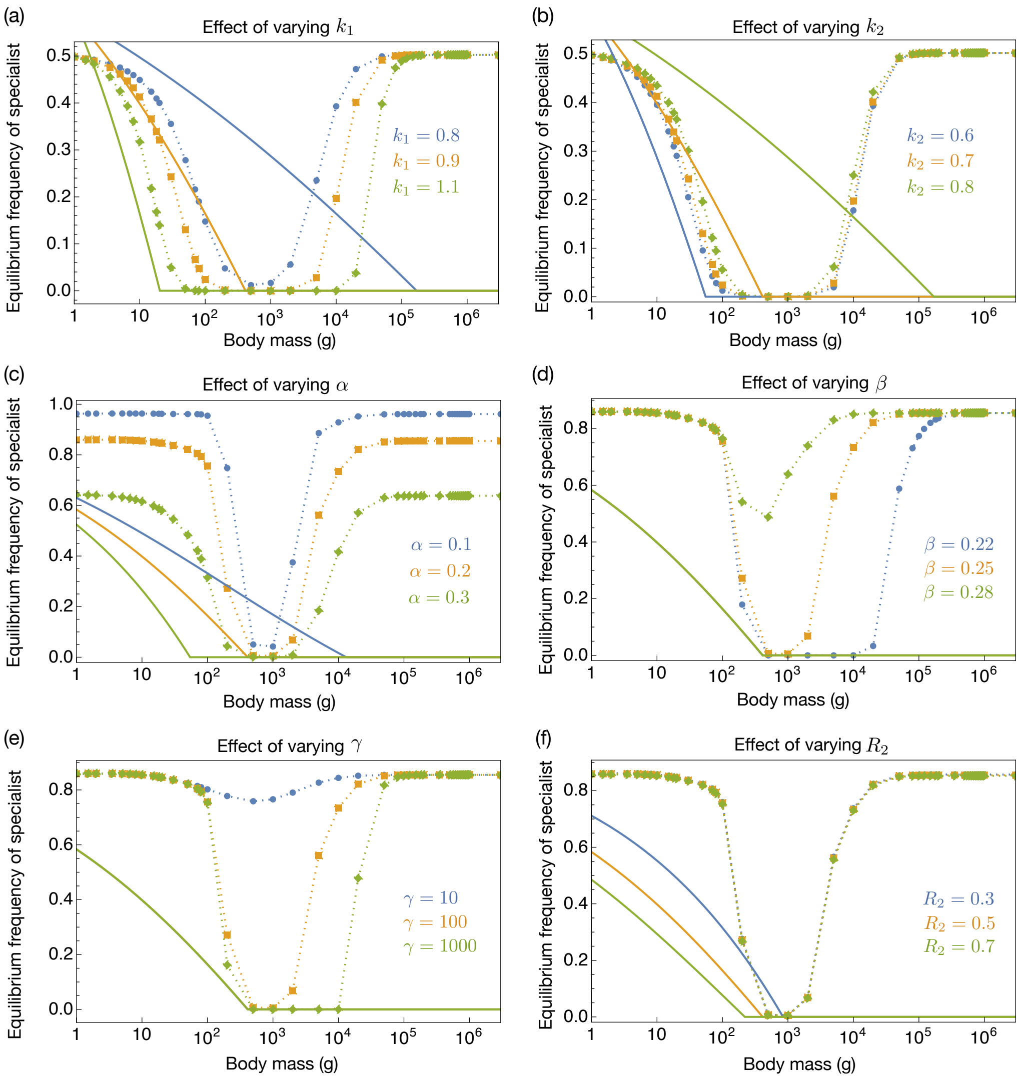

Fig. S12 Frequency of the specialist consumer. The data points are generated numerically at $\boldsymbol{t = 10^1}$, which is during transient ecological dynamics. Symbols of different colors connected by dotted lines are generated from the same set of parameter values. The solid lines of the same colors represent analytical solutions of the competition equilibrium. All other settings and parameters are identical to Fig. 4 of the main text.

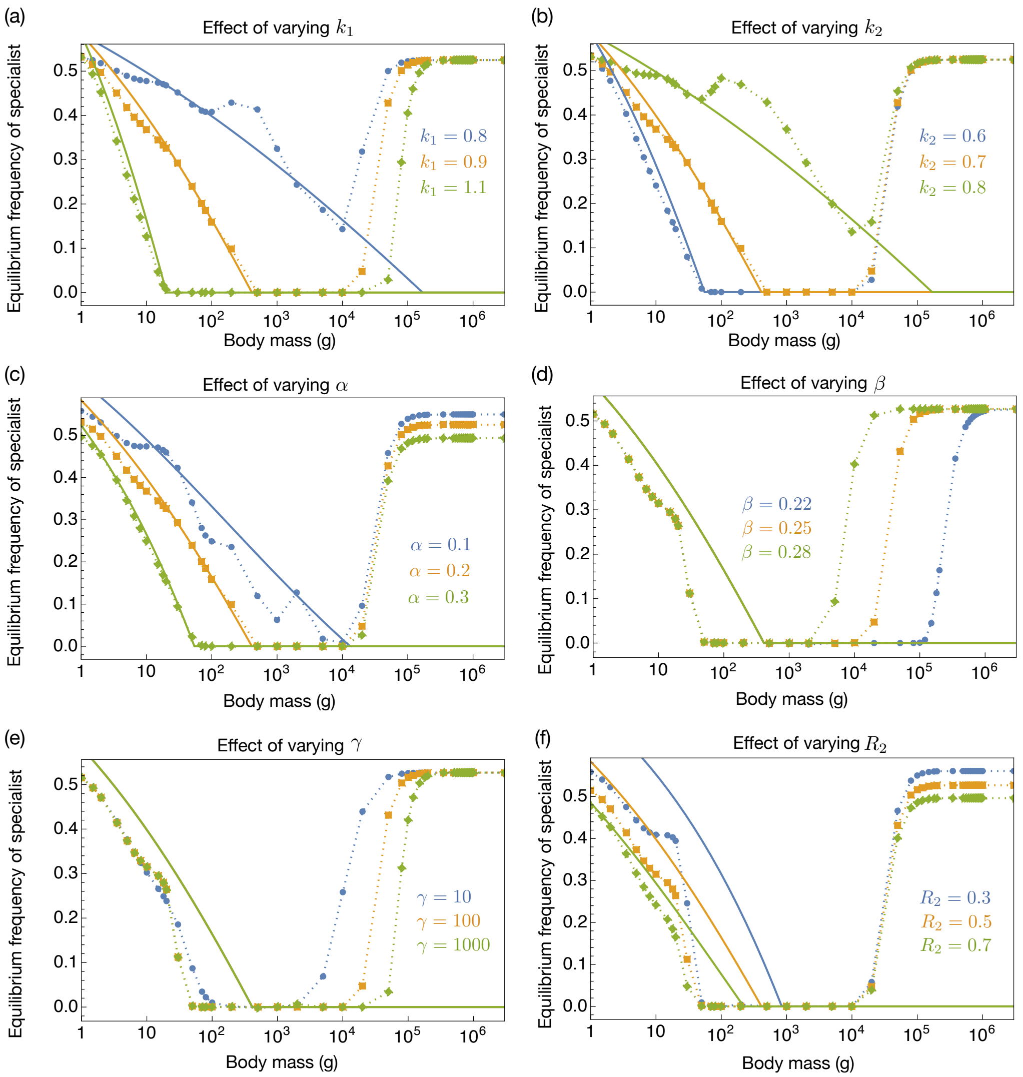

Fig. S13 Frequency of the specialist consumer. The data points are generated numerically at $t = 10^2$, which is during transient ecological dynamics. Symbols of different colors connected by dotted lines are generated from the same set of parameter values. The solid lines of the same colors represent analytical solutions of the competition equilibrium. All other settings and parameters are identical to Fig. 4 of the main text.

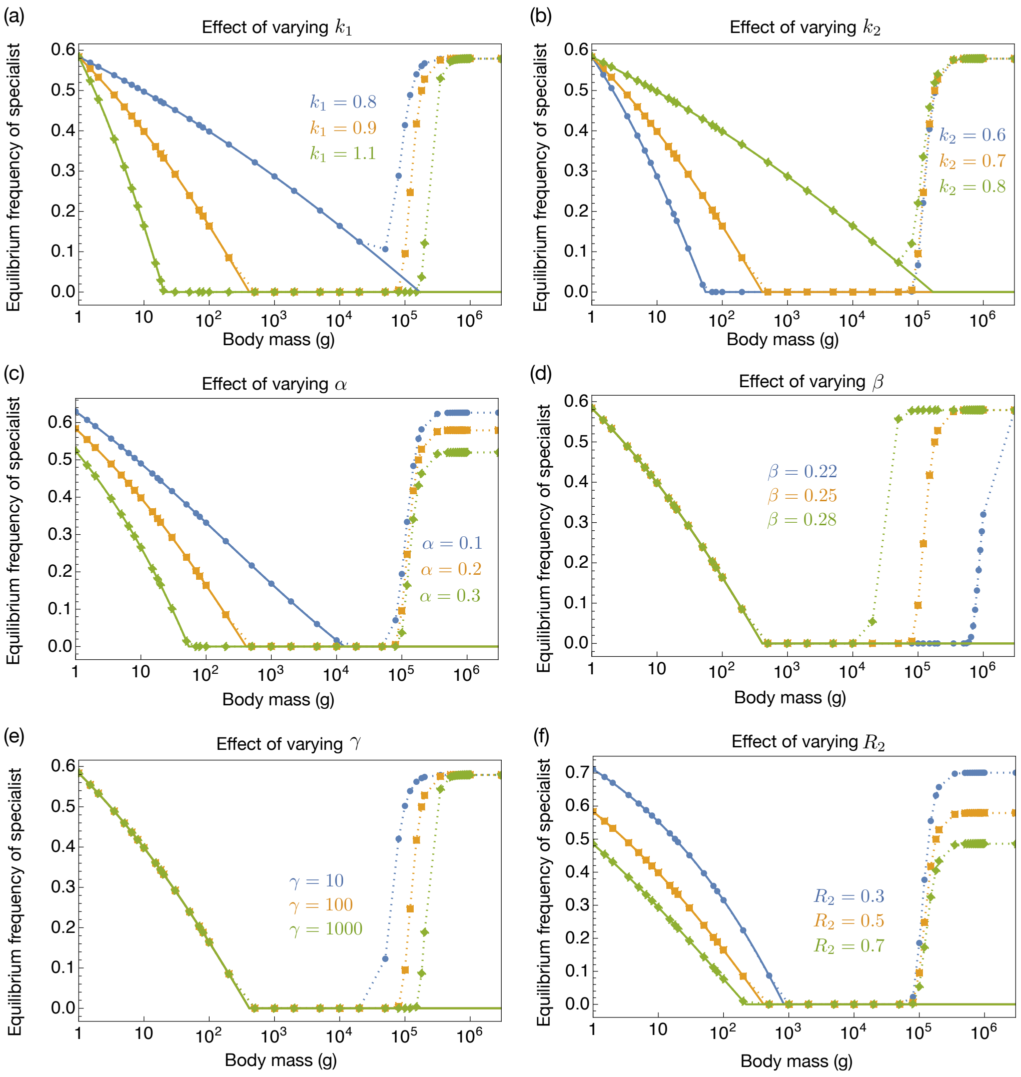

Fig. S14 Frequency of the specialist consumer. The data points are generated numerically at $t = 10^4$. The competition dynamics at small and intermediate body sizes have reached competition equilibrium, while the dynamics at very large body sizes are under quasi-equilibrium. Symbols of different colors connected by dotted lines are generated from the same set of parameter values. The solid lines of the same colors represent analytical solutions of the competition equilibrium. All other settings and parameters are identical to Fig. 4 of the main text.

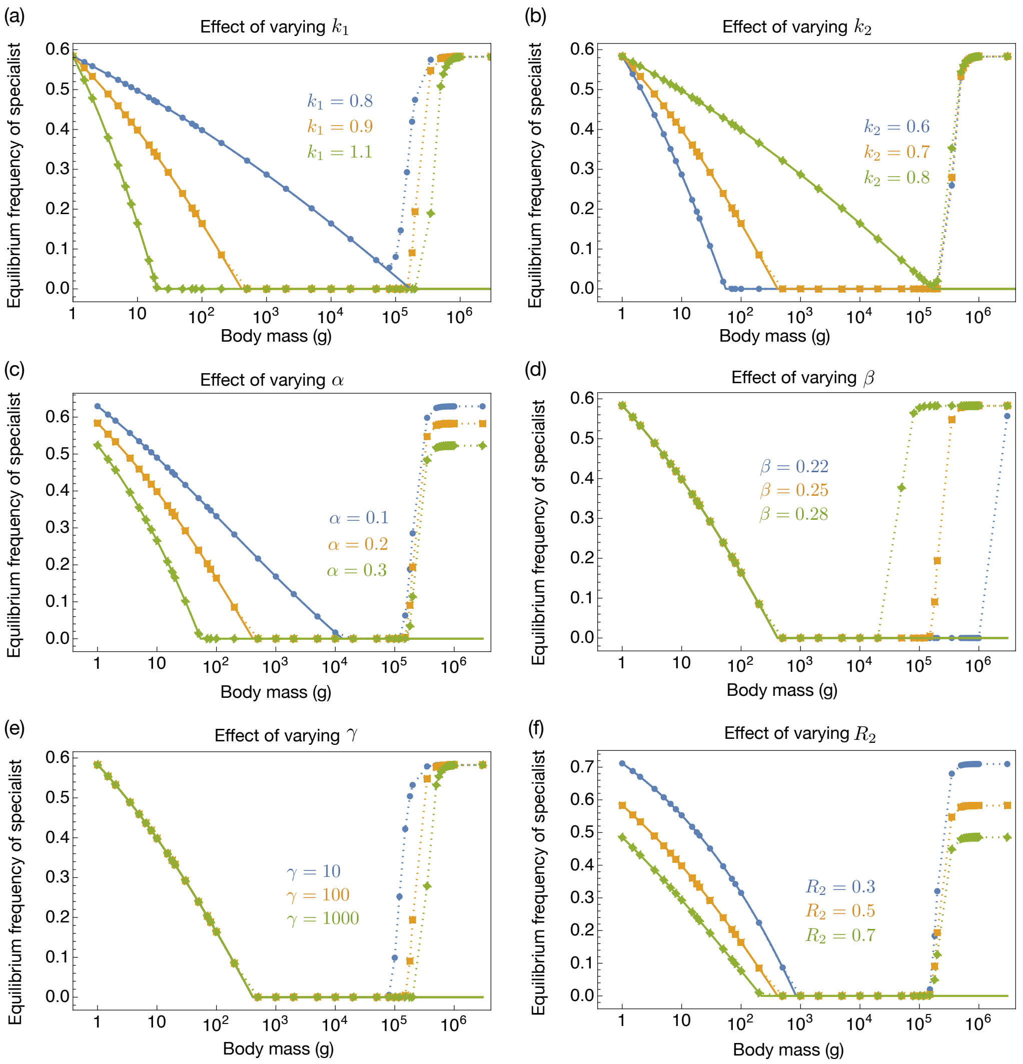

Fig. S15 Frequency of the specialist consumer. The data points are generated numerically at $t = 10^5$. The competition dynamics at small and intermediate body sizes have reached competition equilibrium, while the dynamics at very large body sizes are under quasi-equilibrium. Symbols of different colors connected by dotted lines are generated from the same set of parameter values. The solid lines of the same colors represent analytical solutions of the competition equilibrium. All other settings and parameters are identical to Fig. 4 of the main text.

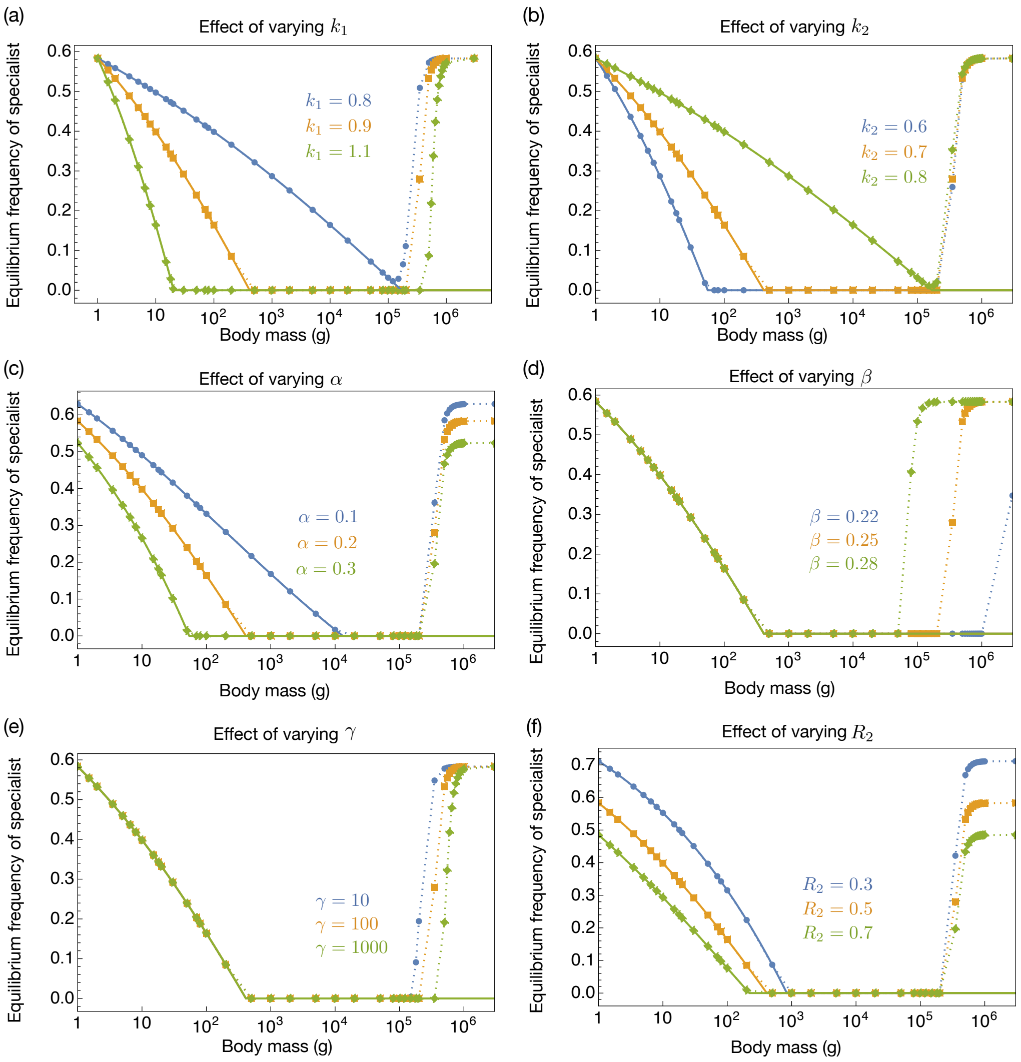

Fig. S16 Frequency of the specialist consumer. The data points are generated numerically at $t = 10^6$. The competition dynamics at small and intermediate body sizes have reached competition equilibrium, while the dynamics at very large body sizes are under quasi-equilibrium. Symbols of different colors connected by dotted lines are generated from the same set of parameter values. The solid lines of the same colors represent analytical solutions of the competition equilibrium. All other settings and parameters are identical to Fig. 4 of the main text.

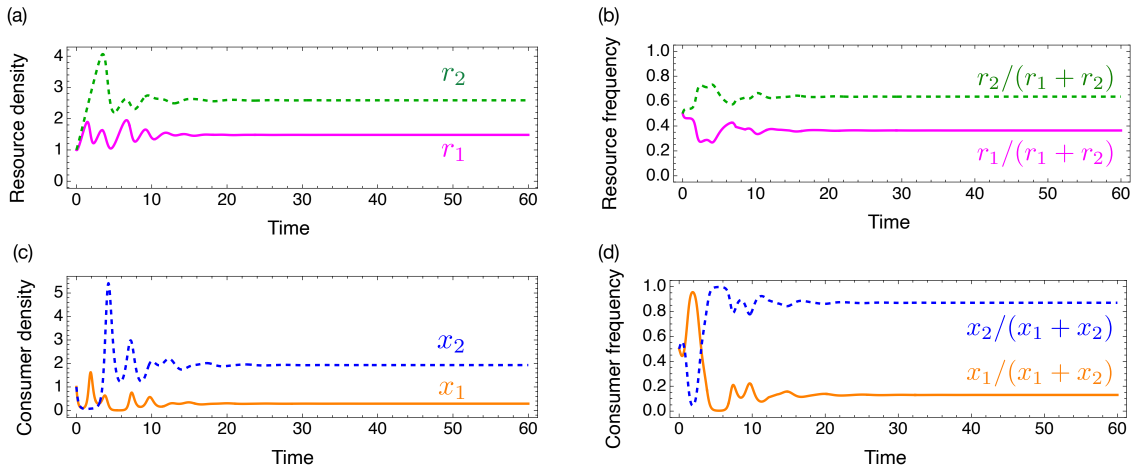

Fig. S17 An example of a numerical solution of the system of ordinary differential equations describing the temporal dynamics of the availability of resources and the abundances of consumers. The body mass of the consumers are $b = 20$g. Other parameter values are $R_1 = R_2 = 1$, $g = 1$, $k_1 = 0.9$, $k_2 = 0.7$, $\alpha = 0.2$, $\beta = 0.2$, $\gamma = 100$, $\kappa = 10$.

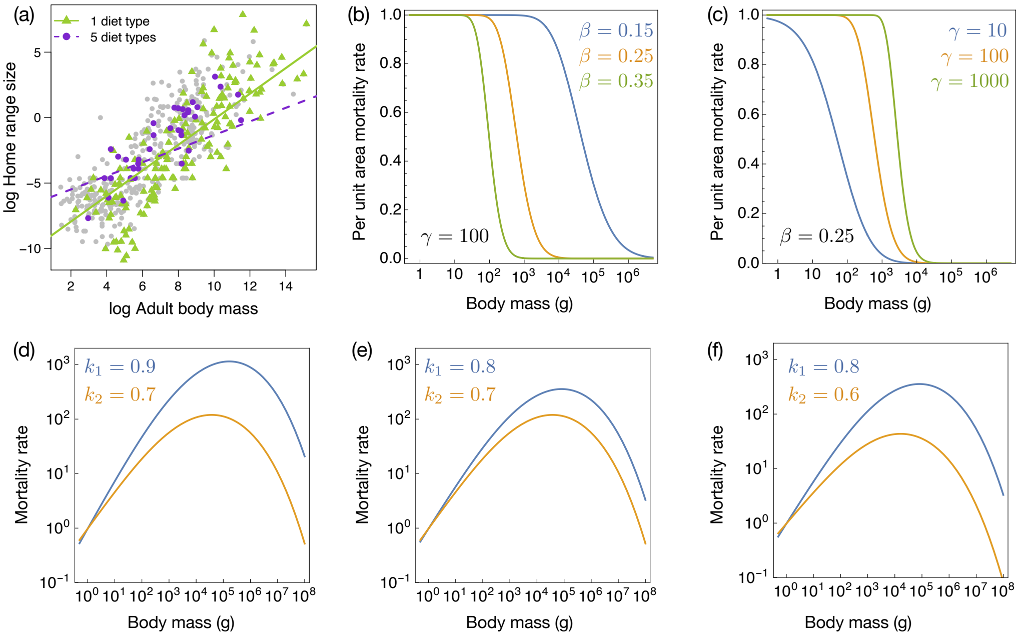

Fig. S18 In animals, (a) the home range size generally increases with adult body mass following a power law, with the scaling exponent for specialists (i.e., 1 diet type) larger than that for generalists (i.e., 5 diet types). (b) The effect of varying parameter β while keeping γ fixed on the per unit area mortality rate as a function of body mass. (c) The effect of varying parameter γ while keeping β fixed on the per unit area mortality rate as a function of body size. (d-f) The overall mortality rate function has a maximum at intermediate body mass for both the generalist and specialist consumers.

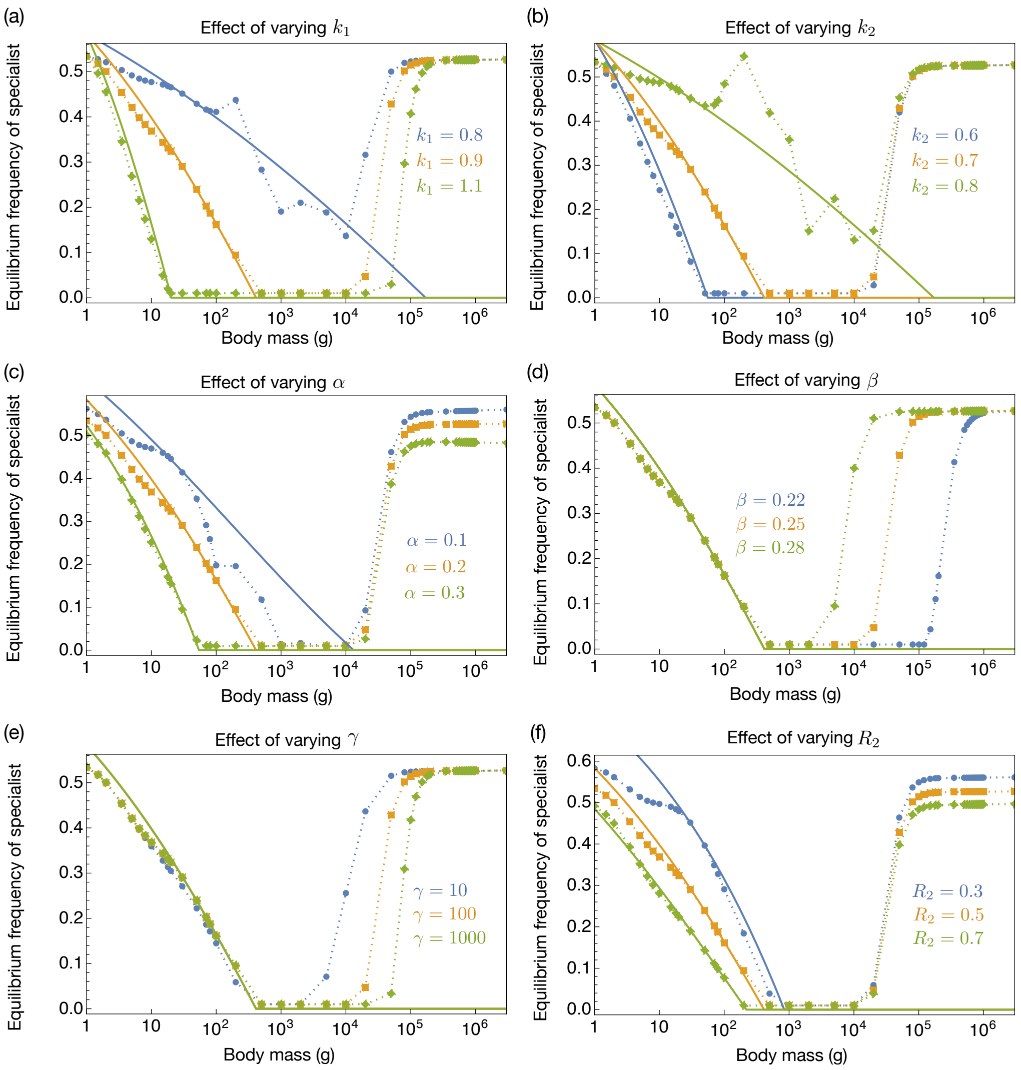

Fig. S19 Frequency of the specialist consumer. The conversion efficiency from food to offspring κ depends on body mass following eq. (12) of the main text, with additional parameters $b_0 = 0.75$, $b_1 = -0.25$, $C_0 = 6$, $C_1 = 0.2$. The data points are generated numerically at $\boldsymbol{t = 10^2}$. All other settings and parameters are identical to Fig. 4 of the main text.

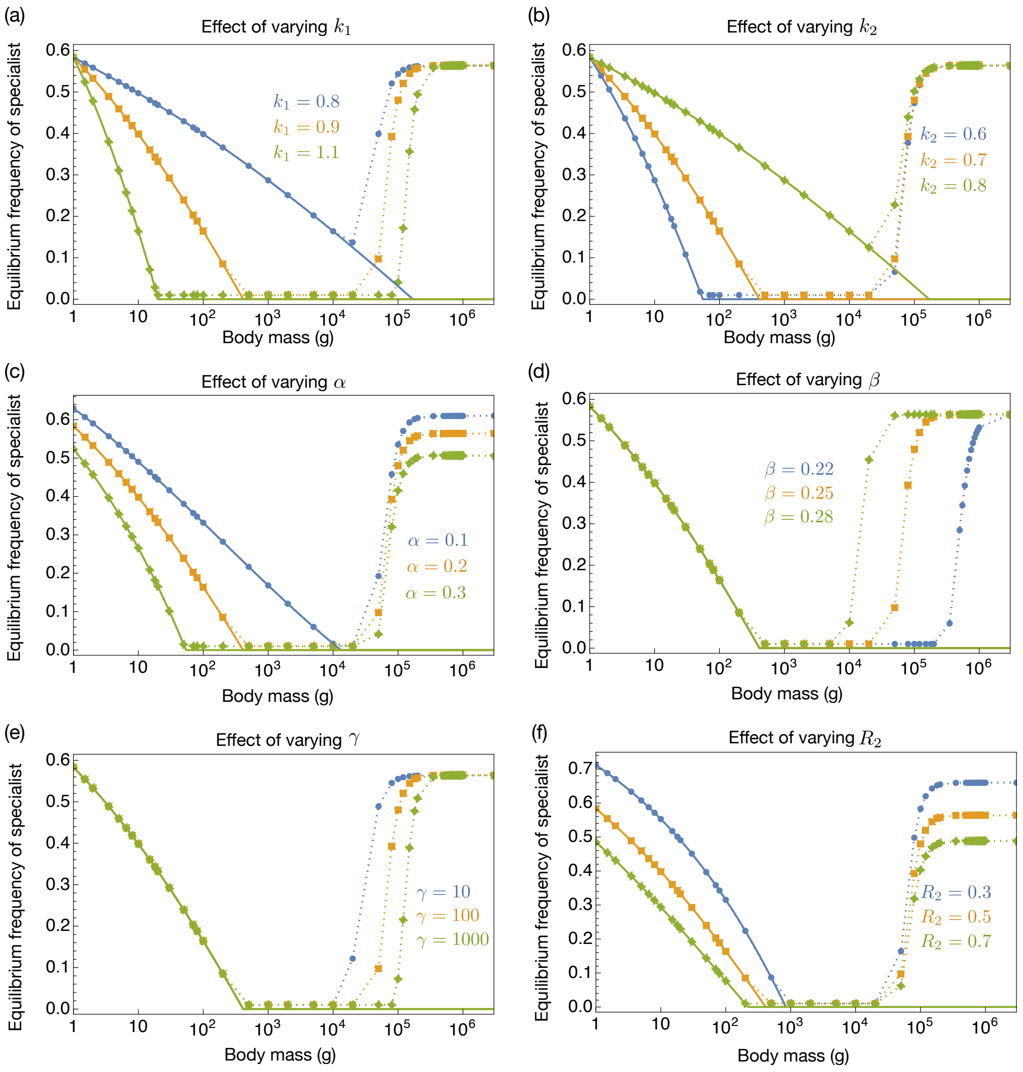

Fig. S20 Frequency of the specialist consumer. The conversion efficiency from food to offspring κ depends on body mass following eq. (12) of the main text, with additional parameters $b_0 = 0.75$, $b_1 = -0.25$, $C_0 = 6$, $C_1 = 0.2$. The data points are generated numerically at $\boldsymbol{t = 10^3}$. All other settings and parameters are identical to Fig. 4 of the main text.

4 Supplementary References

References

[1] Muñoz-Garcia, A. & Williams, J. B. Basal metabolic rate in carnivores is associated with diet after controlling for phylogeny. *Physiological and biochemical Zoology* **78**, 1039–1056 (2005).

[2] Bininda-Emonds, O. R. P. *et al.* The delayed rise of present-day mammals. *Nature* **446**, 507–512 (2007).

[3] Fritz, S. A., Bininda-Emonds, O. R. P. & Purvis, A. Geographical variation in predictors of mammalian extinction risk: Big is bad, but only in the tropics. *Ecology Letters* **12**, 538–549 (2009).

[4] Huang, S., Tucker, M. A., Hertel, A. G., Eyres, A. & Albrecht, J. Scale-dependent effects of niche specialisation: The disconnect between individual and species ranges. *Ecology Letters* **24**, 1408–1419 (2021).

[5] Upham, N. S., Esselstyn, J. A. & Jetz, W. Inferring the mammal tree: Species-level sets of phylogenies for questions in ecology, evolution, and conservation. *PLOS Biology* **17**, e3000494 (2019).

[6] Holt, B. G. *et al.* An update of wallace's zoogeographic regions of the world. *Science* **339**, 74–78 (2013).

[7] Wilman, H. *et al.* EltonTraits 1.0: Species-level foraging attributes of the world's birds and mammals. *Ecology* **95**, 2027–2027 (2014).
