# Supplementary material for: Diverging selection on body size in specialist terrestrial mammals
Source: Nat Ecol Evol. 2026 Jan 28;10(2):342–54. doi: 10.1038/s41559-025-02959-2 (PMC12890589; doi:10.1038/s41559-025-02959-2)
Supplement: Supplementary file 2 — Reporting Summary [file 41559_2025_2959_MOESM2_ESM.pdf]

nature portfolio

Corresponding author(s): Ziyue Chen

Last updated by author(s): Nov 28, 2025

Reporting Summary

Nature Portfolio wishes to improve the reproducibility of the work that we publish. This form provides structure for consistency and transparency in reporting. For further information on Nature Portfolio policies, see our Editorial Policies and the Editorial Policy Checklist.

Statistics

For all statistical analyses, confirm that the following items are present in the figure legend, table legend, main text, or Methods section.

n/a	Confirmed	
☐	☒	The exact sample size (*n*) for each experimental group/condition, given as a discrete number and unit of measurement
☐	☒	A statement on whether measurements were taken from distinct samples or whether the same sample was measured repeatedly
☐	☒	The statistical test(s) used AND whether they are one- or two-sided<br>*Only common tests should be described solely by name; describe more complex techniques in the Methods section.*
☐	☒	A description of all covariates tested
☐	☒	A description of any assumptions or corrections, such as tests of normality and adjustment for multiple comparisons
☐	☒	A full description of the statistical parameters including central tendency (e.g. means) or other basic estimates (e.g. regression coefficient) AND variation (e.g. standard deviation) or associated estimates of uncertainty (e.g. confidence intervals)
☐	☒	For null hypothesis testing, the test statistic (e.g. *F*, *t*, *r*) with confidence intervals, effect sizes, degrees of freedom and *P* value noted<br>*Give P values as exact values whenever suitable.*
☒	☐	For Bayesian analysis, information on the choice of priors and Markov chain Monte Carlo settings
☒	☐	For hierarchical and complex designs, identification of the appropriate level for tests and full reporting of outcomes
☐	☒	Estimates of effect sizes (e.g. Cohen's *d*, Pearson's *r*), indicating how they were calculated

Our web collection on statistics for biologists contains articles on many of the points above.

Software and code

Policy information about availability of computer code

Data collection	No software was used.
Data analysis	1. The calculation of CES-D 10 scores was based on CHARLS data and conducted using Python 3.8 (https://www.python.org/).<br>2. The preprocessing of historical sunlight duration station data, spatial interpolation (Thin Plate Spline method), and anomaly detection and correction for the time series were performed using ArcGIS 10.4 and Python 3.8 (https://www.python.org/).<br>3. The development of high-dimensional fixed effects and ordinal logistic regression models to analyze the relationship between CES-D 10 scores and historical sunlight duration was carried out in Python 3.8 (https://www.python.org/).<br>4. The prediction of sunlight duration across RCP–SSP scenarios using MLP models, along with the projection of mental health outcomes for older adults in China under different Shared Socioeconomic Pathways and the estimation of future annual per-capita incremental medical expenditures related to mental health, were all implemented in Python 3.8 (https://www.python.org/).<br>5. Plots were generated with Python 3.8 (https://www.python.org/).

For manuscripts utilizing custom algorithms or software that are central to the research but not yet described in published literature, software must be made available to editors and reviewers. We strongly encourage code deposition in a community repository (e.g. GitHub). See the Nature Portfolio guidelines for submitting code & software for further information.

April 2023

Data

Policy information about availability of data

All manuscripts must include a data availability statement. This statement should provide the following information, where applicable:

- Accession codes, unique identifiers, or web links for publicly available datasets
- A description of any restrictions on data availability
- For clinical datasets or third party data, please ensure that the statement adheres to our policy

1. The multi-wave data from CHARLS are available at https://opendata.pku.edu.cn/dataverse/CHARLS.
2. Historical sunlight duration data from China's surface meteorological stations can be accessed at https://www.resdc.cn/data.aspx?DATAID=230.
3. Future SDSR, TCC, and TOA data from the FGOALS-g3 model are available at DOI: 10.24381/cds.c866074c.
4. Meteorological indicators were sourced from the National Centers for Environmental Information (NCEI) under the National Oceanic and Atmospheric Administration (NOAA) and the Modern-Era Retrospective analysis for Research and Applications, Version 2 (MERRA-2) (https://gmao.gsfc.nasa.gov/gmao-products/merra-2/data-access_merra-2/?utm_source=chatgpt.com).
5. Air pollution data are available at https://zenodo.org/records/10472666?utm_source=chatgpt.com.
6. City-level GDP per capita were derived from the China City Statistical Yearbook (2010-2018) (https://www.stats.gov.cn/).
7. The NDVI datasets are available from https://www.earthdata.nasa.gov/data/catalog/lpcloud-mod13a3-061?utm_source=chatgpt.com.

Research involving human participants, their data, or biological material

Policy information about studies with human participants or human data. See also policy information about sex, gender (identity/presentation), and sexual orientation and race, ethnicity and racism.

Reporting on sex and gender	Sex/gender was determined based on self-reporting in the CHARLS survey. The study included both male and female respondents. Gender was used as a covariate in the statistical models (e.g., Equation 1 and Table A1) and in heterogeneity analysis to examine differences in mental health outcomes.
Reporting on race, ethnicity, or other socially relevant groupings	The study utilizes data from CHARLS, covering residents across 31 provinces in China. Race or ethnicity was not used as a primary categorization variable in this specific analysis focusing on climate and sunlight exposure.
Population characteristics	The study focuses on Chinese adults aged 45 and above. The final analytical sample includes 14,910 primary respondents. Key covariate-relevant characteristics include age, gender, hukou status (urban/rural), marital status, social activity levels, and mental health status measured by the CES-D 10 scale.
Recruitment	Participants were recruited through the CHARLS national baseline and follow-up surveys, which employed a multistage, stratified probability-proportional-to-size (PPS) sampling method to ensure national representativeness. Potential self-selection bias is minimized by the randomized sampling design of the original survey.
Ethics oversight	The original CHARLS study was approved by the Ethical Review Committee of Peking University. As this study utilizes de-identified secondary data, additional ethical approval was not required, but the original ethical standards were adhered to.

Note that full information on the approval of the study protocol must also be provided in the manuscript.

Field-specific reporting

Please select the one below that is the best fit for your research. If you are not sure, read the appropriate sections before making your selection.

☐ Life sciences ☒ Behavioural & social sciences ☐ Ecological, evolutionary & environmental sciences

For a reference copy of the document with all sections, see nature.com/documents/nr-reporting-summary-flat.pdf

Behavioural & social sciences study design

All studies must disclose on these points even when the disclosure is negative.

Study description	This study conducted a longitudinal analysis by linking over 50,000 observations of older adults from the China Health and Retirement Longitudinal Study (CHARLS) to high-resolution, daily sunlight duration data from more than 2,400 meteorological stations. We applied semi-parametric temperature-bin ordinal logistic regressions and panel fixed-effects models to characterize the potentially non-linear associations between daily sunlight duration and depressive symptoms among older adults. Recognizing the limited attentions given to the impact of future climate-induced changes in sunlight availability, we specifically integrated atmospheric physics-based climate models with advanced neural network approaches to project future sunlight duration across China under different Shared Socioeconomic Pathways (SSPs). Based on the projection, we further estimated the corresponding incremental healthcare expenditures.
Research sample	The CHARLS survey encompassed over 28,000 individuals from 31 provinces (excluding Tibet), 150 counties/districts, and 450 villages/communities, generating 112,879 questionnaire records. The study incorporated data from four waves of CHARLS conducted

	in 2011, 2013, 2015, and 2018. Focusing specifically on data from 14,910 primary respondents, excluding data from respondents' relatives to avoid potential correlation bias. The samples are nationally representative.
Sampling strategy	CHARLS employed a multistage, stratified, PPS (probability proportional to size) sampling method. Initially, 150 county-level units (including urban districts and counties) were randomly selected nationwide. Subsequently, three villages/communities were randomly chosen within each county-level unit, followed by the random selection of households within each village/community as samples.
Data collection	The datasets we use are existing data and are open source, including CHARLS, Historical sunlight duration data, Future SDSR, TCC, and TOA data, etc. See the data availability statement for data acquisition.
Timing	The CHARLS survey data and historical daily sunlight duration data cover the years 2011, 2013, 2015, and 2018, with daily sunlight duration measured at a precision of 0.1 hours. Future climate data derived from the FGOALS-g3 model span the period from 2021 to 2100.
Data exclusions	The survey encompassed more than 28,000 individuals, and the study focused on 14,910 primary respondents. We excluded data from respondents' relatives (spouses/children) to avoid potential correlation bias and ensure independent observations. Data from the year 2020 were also excluded to avoid the anomalous impact of the COVID-19 pandemic.
Non-participation	No participants dropped out/declined participation.
Randomization	The samples in the CHARLS dataset were randomly selected and ensured national representativeness through a multistage, stratified, probability-proportional-to-size (PPS) sampling method.

Reporting for specific materials, systems and methods

We require information from authors about some types of materials, experimental systems and methods used in many studies. Here, indicate whether each material, system or method listed is relevant to your study. If you are not sure if a list item applies to your research, read the appropriate section before selecting a response.

Materials & experimental systems

n/a	Involved in the study
☒	☐ Antibodies
☒	☐ Eukaryotic cell lines
☒	☐ Palaeontology and archaeology
☒	☐ Animals and other organisms
☒	☐ Clinical data
☒	☐ Dual use research of concern
☒	☐ Plants

Methods

n/a	Involved in the study
☒	☐ ChIP-seq
☒	☐ Flow cytometry
☒	☐ MRI-based neuroimaging

Plants

Seed stocks	*Report on the source of all seed stocks or other plant material used. If applicable, state the seed stock centre and catalogue number. If plant specimens were collected from the field, describe the collection location, date and sampling procedures.*
Novel plant genotypes	*Describe the methods by which all novel plant genotypes were produced. This includes those generated by transgenic approaches, gene editing, chemical/radiation-based mutagenesis and hybridization. For transgenic lines, describe the transformation method, the number of independent lines analyzed and the generation upon which experiments were performed. For gene-edited lines, describe the editor used, the endogenous sequence targeted for editing, the targeting guide RNA sequence (if applicable) and how the editor was applied.*
Authentication	*Describe any authentication procedures for each seed stock used or novel genotype generated. Describe any experiments used to assess the effect of a mutation and, where applicable, how potential secondary effects (e.g. second site T-DNA insertions, mosiacism, off-target gene editing) were examined.*
